# Supplementary material for: Domain-Swapped LuxR-Type Quorum Sensing Receptors Reveal Divergent Ligand-Response Mechanisms among Homologues
Source: J Am Chem Soc. 2026 Aug 27;148(35):38256–70. doi: 10.1021/jacs.6c11050 (PMC13564444; doi:10.1021/jacs.6c11050)
Supplement: Supplementary file 1 [file ja6c11050_si_001.pdf]

SUPPORTING INFORMATION

Domain-swapped LuxR-type quorum sensing receptors reveal divergent ligand-response mechanisms among homologs

Irene M. Stoutland,[a] Susan A. Walker,[a] and Helen E. Blackwell[a,]*

[a]*Department of Chemistry, University of Wisconsin–Madison, 1101 University Ave., Madison, WI, 53706 USA*

*Address correspondence to Helen E. Blackwell, blackwell@chem.wisc.edu.

CONTENTS

- Supplemental methods
- Discussion of effect of FLAG tag on reporter activity
- **Figure S1.** Sequence alignment of selected LuxR-type receptors
- **Figure S2.** Sequence alignment comparison of CinR vs. MrtR and CinI vs. MrtI
- **Figure S3.** Response of chimeras to ligands corresponding to the LBD vs. DBD receptor
- **Figure S4.** Additional chimera and mutant activity data
- **Figure S5.** Effects of FLAG tag on activity
- **Figure S6.** Activity of DBDs alone
- **Figure S7.** Ligand potency in EsaR and ExpR2 linker deletion mutants
- **Figure S8.** Activity and accumulation of FLAG-tagged EsaR linker addition mutants
- **Figure S9.** Small molecule inhibition of select mutants and chimeras
- **Figure S10.** Quantile-Quantile plots
- **Figure S11.** Optimization of arabinose concentration for *E. coli* BW27749 reporters
- **Figure S12.** Additional biological replicate of western blots
- **Table S1.** Protein sequences of WT LuxR-type receptors examined in this study
- **Table S2.** Bacterial strains and plasmids used in this study
- **Table S3.** Ordinary one-way ANOVA with Šídák's correction for multiple comparisons for activity of EsaR +5 linker mutants with DMSO, corresponding to data in **Figure 5F**
- **Table S4.** Ordinary one-way ANOVA with Šídák's correction for multiple comparisons for activity of ExpR2 +5 linker mutants with DMSO, corresponding to data in **Figure 5G**
- **Table S5.** Ordinary one-way ANOVA with Šídák's correction for multiple comparisons for activity of MrtR +4 linker mutants with 7Z-3OHC14, corresponding to data in **Figure 5B**
- **Table S6.** gBlocks used in this study
- **Table S7.** Primers used in this study
- Raw western blot images
- References

Supplemental methods.

Western blots. Cultures were grown as described for bacterial reporters, either in 96-well microtiter plates or in test tubes. The OD_{600} of each sample was measured using a plate reader, and X mL of culture was pelleted, where X = 0.25/OD_{600}. Cell pellets were resuspended in 100 µL MilliQ water and stored at -20 °C until SDS-PAGE analysis (see SDS-PAGE protocol below). Gels were equilibrated in transfer buffer for 20 min and trimmed to a 15-70 kDa range before transfer. PVDF membrane (Immobilon-P) was soaked in methanol for 30 s to 1 min, then membrane, filter paper, and sponges were equilibrated in chilled transfer buffer (10 mM CAPS, 10% methanol, pH 10-11) for 20 min. Transfer was performed using a Bio-Rad Mini-PROTEAN Tetra Cell, with ice packs and on a stir plate, for 120 min at 40V. Blots were stained immediately or stored at 4 °C overnight between sheets of wet filter paper. Blots were stained with HRP-conjugated DYKDDDDK Tag Monoclonal antibody from Proteintech (Cat No. HRP-66008) to visualize FLAG-tagged proteins, or with HRP-conjugated GAPDH Monoclonal antibody from Proteintech (Cat No. HRP-66004) to visualize GAPDH as a loading control. In both cases, blots were blocked (EveryBlot Blocking Buffer, Bio-Rad) for 20 min, and then antibodies were added at a 1:15,000 dilution for 50 min to 1 hr. Blots were washed with TBST (20 mM Tris base, 150 mM NaCl, 0.1% Tween 20) once for 10 sec, then three times for 5-10 min. Blots were visualized with Radiance Plus (Azure Biosystems) on an Azure Biosystems C400 imager. After imaging, blots were incubated with 10% acetic acid for 30 min at 37 °C to inactivate HRP, then re-probed for the loading control. Alternatively, duplicate blots were performed for FLAG and GAPDH staining. Brightness and contrast of images was adjusted to improve visibility of bands, and no non-linear corrections were applied. Raw images are shown at the end of this SI document.

SDS-PAGE for western blot and solubility assays. Samples were prepared with 12 µL sample, 4.5 µL of 4x LDS buffer (G-Biosciences cat. #786323), and 1.5 µL of 20 mM DTT, then heated to 95 °C for 10 min before loading on a 10% Mini-PROTEAN TGX gel (Bio-Rad). Gels were run on ice for 100 min at 120V. Gels were used for western blots, as described above, or imaged using a Cytiva Amersham Typhoon imager for solubility assays.

Solubility assay. Proteins were expressed from *E. coli* BL21(DE3) harboring pLysS and a pET17b plasmid expressing the protein of interest. pET17b-FLAG-lasR was constructed by inserting a FLAG tag between LasR residues 1 and 2 in pET17b-lasR. Overnight cultures were diluted 1:100 in LB medium with 100 µg/mL ampicillin and 34 µg/mL chloramphenicol and grown at 37 °C with 200 rpm shaking to an OD_{600} of 0.5-0.7, as measured using a cuvette on a Nanodrop 2000c spectrophotometer (Thermo Scientific). Cultures were chilled on ice for 10 min and supplemented with 0.5 µM IPTG to induce protein expression. Compound was added as a 1:1000 dilution of a 10 mM stock of 3OC12 in DMSO, and an equivalent volume of DMSO was added to control cultures. Cultures were incubated at 17 °C with shaking at 200 rpm for 16 hr. Cells were pelleted by centrifugation, resuspended in TEDG buffer (50 mM Tris HCl, 500 mM NaCl, 1 mM EDTA, 10% glycerol, 1 mM DTT, pH 7.4, supplemented with 10 µM 3OC12 or DMSO and a protease inhibitor cocktail) and lysed by sonication. Samples were taken of the whole cell lysate, then the lysate was centrifuged at 40,000g for 30 min. The supernatant was collected as the soluble fraction. Whole cell and soluble fractions were stored at -20 °C until analysis by SDS-PAGE.

Sequence alignment generation. Sequences from the National Center for Biotechnology Information (NCBI) database were aligned using Clustal Omega and visualized with MView.[1, 2]

Discussion of effect of FLAG tag on reporter activity.

To aid in visualization for western blotting, a FLAG tag was added to the N-terminus of each protein. In some cases, the addition of a FLAG or FLAG-Gly5 tag to a receptor affected its activity in a transcriptional reporter. EsaR was inactive with a FLAG tag, likely because the tag interfered with far N-terminal interactions (**Figure 6A**, **C**, and **D**) but partially active when a Gly-5 linker was added after the FLAG tag (**Figure S5A** and **B**), so a FLAG-Gly5 tag was used for chimeras with the EsaR N-terminus. The addition of an N-terminal FLAG tag to WT LasR or a FLAG-Gly5 tag to chimeras with a LasR DBD dramatically increased activity in reporter assays (**Figure S5C** and **D**). Other tags also caused large changes in LasR activity in the reporter assay (**Figure 6F**). We considered that the FLAG tag may have a stabilizing effect on LasR in the absence of ligand, but a solubility assay showed that, like WT LasR, FLAG-LasR is soluble in the presence of 3OC12 but not in its absence (**Figure S5F**).

Alternatively, the presence of an N-terminal tag may alter protein expression by affecting mRNA folding around the translation initiation region (TIR).[3, 4] Higher protein expression would be expected to lead to higher activity in a transcriptional reporter assay. Notably, although WT LasR and chimeras with a LasR DBD differ in LBD, their respective protein production plasmids have the same sequence immediately upstream of the translational start site, and this sequence is slightly different from the plasmid expressing EsaR and chimeras with an EsaR DBD. Chimeras were constructed so that the backbone matched the backbone of the WT plasmid to which they were compared in reporter assays. While chimera EL-10 expressed from the same backbone as LasR showed a 13-fold increase in activity upon the addition of a FLAG tag, EL-10 expressed from that plasmid with 12 bp removed upstream of the start codon showed ~9-fold higher activity than EL-10 expressed from the original plasmid and showed a slight *decrease* in activity upon the addition of a FLAG tag (**Figure S5G**). These results support the hypothesis that tag-induced changes in LasR activity are due to changes in the TIR, rather than changes to protein activity.

In some cases, the FLAG tag also affected ligand responsiveness. While chimeras EL-3 and EL-6 showed decreased activity in the presence of 3OC6, FLAG-Gly5-EL-3 and FLAG-Gly5-EL-6 showed much higher overall signal than their untagged counterparts and no response to ligand. A very high level of protein expression could overwhelm the detection limit of the reporter system, causing receptors to appear constitutively active. To test the effects of protein expression level on ligand responsiveness, we expressed FLAG-Gly5-EL-3 and FLAG-Gly5-EL-6 in an *E. coli* BW27749 reporter strain in which protein expression could be controlled based on the concentration of arabinose used for induction. Decreasing arabinose concentrations led to a dramatic decrease in overall signal but did not restore ligand responsiveness in FLAG-Gly5-EL-3 and FLAG-Gly5-EL-6 (**Figure S5H-I**), suggesting that the lack of ligand response is not an artifact of protein expression levels.

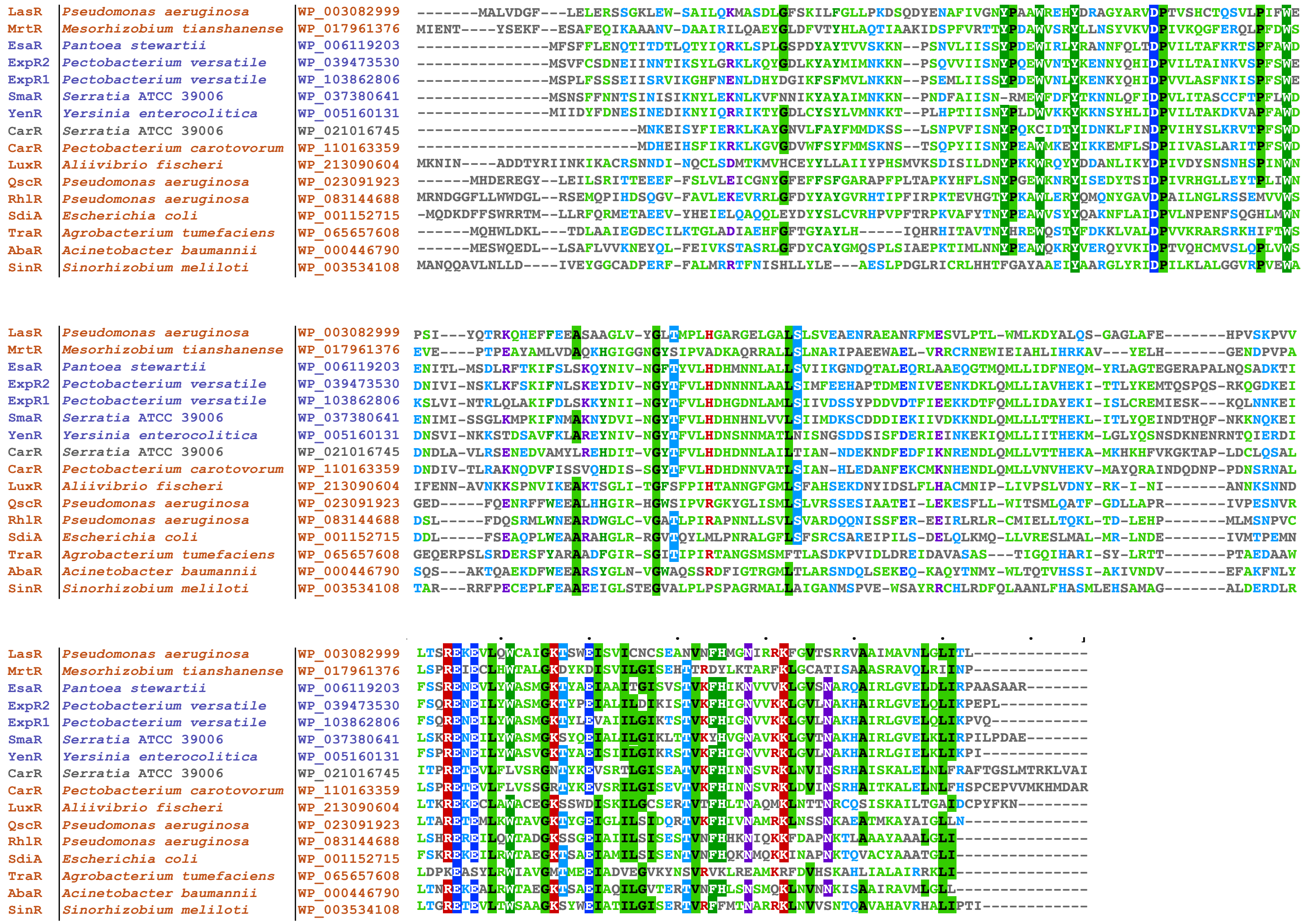

Figure S1. Sequence alignment of selected LuxR-type receptors. Sequences shown in purple are dissociative,[5-8] those in orange are associative,[9-14] and CarR (gray) is unresponsive to ligand.[11] Coloring indicates shared properties of aligned residues: polar (light blue); charged (purple); positive charge (red); negative charge (dark blue); hydrophobic, aliphatic, small, or turn-like (light green); aromatic (dark green); not conserved (gray). Separate instances of the same amino acid may have different coloring to highlight different shared properties. Highlighted residues indicate high conservation. Alignment created with Clustal Omega and visualized with MView.[1, 2]

A

```
AAL59595.1_CinR_R_etli      MIENTYSDKFEPAFEQIKAAPNVDAAIRILQAEYGLDFVTYHLAQTIASKIDSPFVRTTY
MrtR                        MIENTYSEKFESAFEQIKAAANVDAAIRILQAEYGLDFVTYHLAQTIAAKIDSPFVRTTY
                            *******:*** ******** ***************************:***********

AAL59595.1_CinR_R_etli      PDAWVSRYLLNSYVKVDPIVKQGFERQLPFDWSEVEPTPEAYAMLVDAQKHGIGGNGYSI
MrtR                        PDAWVSRYLLNSYVKVDPIVKQGFERQLPFDWSEVEPTPEAYAMLVDAQKHGIGGNGYSI
                            ************************************************************

AAL59595.1_CinR_R_etli      PVADKAQRRALLSMNARIPADHWAELVRRCRNEWIEIAHLIHQKAVYELYGENDPVPALS
MrtR                        PVADKAQRRALLSLNARIPAEEWAELVRRCRNEWIEIAHLIHRKAVYELHGENDPVPALS
                            *************:******:.********************:******:**********

AAL59595.1_CinR_R_etli      PREIECLHWTALGKDYKDISVILGISEHTTRDYLKTARFKLGCATISAAASRAVQLRIINP    241
MrtR                        PREIECLHWTALGKDYKDISVILGISEHTTRDYLKTARFKLGCATISAAASRAVQLRIINP    241
                            *************************************************************
```

B

```
AAF89990.1_CinI_R_leguminosarum      MFVIIQAHEYQKYAAVLDQMFRLRKKVFADTLCWDVPVIGPYERDSYDSLAPAYLVWCND
AAZ32755.1_MrtI_M_tianshanense       MFIIIQAHEYQKYAAVLDQMFRLRKKVFADTLGWDVPVIGPYERDSYDSLAPAYLVWCND
                                     **:***************************** ***************************

AAF89990.1_CinI_R_leguminosarum      SRTRLYGGMRLMPTTGPTLLYDVFRETFPDAADLIAPGIWEGTRMCIDEEAIAKDFPEID
AAZ32755.1_MrtI_M_tianshanense       SRTRLYGGMRLMPTTGPTLLYDVFRETFPDAANLIAPGIWEGTRMCIDEEAIAKDFPGID
                                     ********************************:************************ **

AAF89990.1_CinI_R_leguminosarum      AGRAFSMMLLALCECALDHGIHTMISNYEPYLKRVYKRAGAEVEELGRADGYGKYPVCCG
AAZ32755.1_MrtI_M_tianshanense       AGRAFSMMLLALCECALDHGIHTMISNYEPYLKRVYKRAGAEVEELGRADGYGKYPVCCG
                                     ************************************************************

AAF89990.1_CinI_R_leguminosarum      AFEVSDRVLRKMRAALGLTLPLYVRHVPARSVVTQFLEMAA  221
AAZ32755.1_MrtI_M_tianshanense       AFEVSDRVLRKMRAALGLTLPLYVRHVPARSVVTQFLEMAA  221
                                     *****************************************
```

Figure S2. Sequence alignment comparison of CinR vs. MrtR and CinI vs. MrtI. (A) Sequence alignment of CinR of *Rhizobium etli* and MrtR of *Mesorhizobium tianshanense* with matching translational start sites. CinR and MrtR are 96% identical. **(B)** Sequence alignment of AHL synthases CinI of *Rhizobium leguminosarum* and MrtI of *M. tianshanense*. CinI and MrtI are 98% identical. NCBI accession numbers are indicated at the start of each row, except for MrtR, as the chosen translational start site differs from the MrtR sequence available in the NCBI database (AAZ32754.1) but matches non-redundant protein sequence WP_017961376. Alignments created with Clustal Omega.[1, 2]

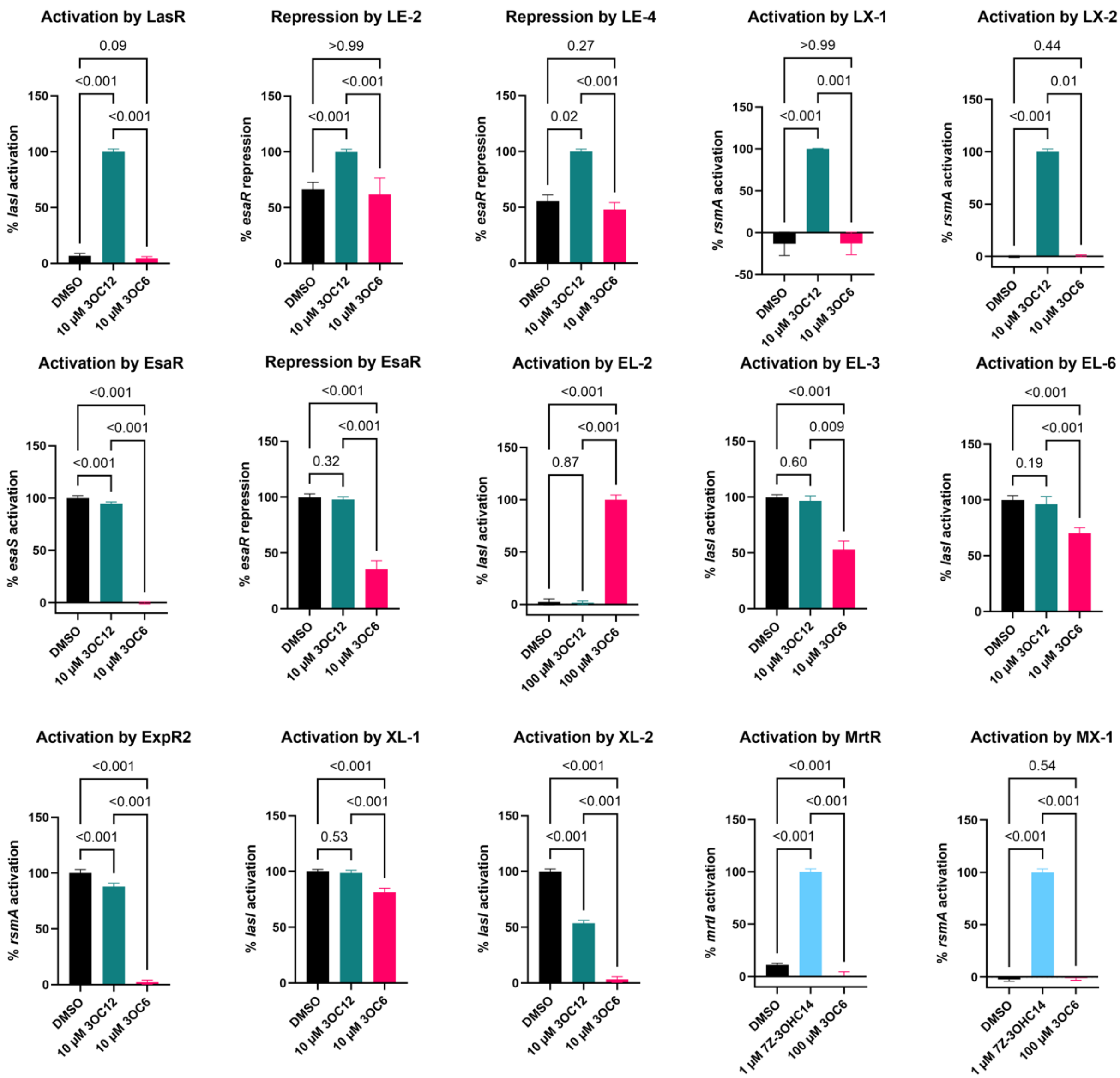

Figure S3. Response of chimeras to ligands corresponding to the LBD vs. DBD receptor. All data were collected in the same *E. coli* JLD271 reporter strains as the data shown in **Figure 4**. Data are normalized to uninduced culture (0%) and the maximum activity of each construct (100%). Data represent at least three biological replicates, each performed in technical triplicate, except for the "Activation by MrtR" data, which represents two biological replicates. **(All graphs)** Error bars indicate standard deviation. For plots with data that passed the Shapiro-Wilk test for normality and the Brown-Forsythe test for homogeneity of variance, p-values are from an ordinary 1-way ANOVA with the Tukey correction for multiple comparisons (activation by LasR, EsaR, ExpR2, MrtR, EL-2, EL-6, XL-1, XL-2, or MX-1); for data that passed the Shapiro-Wilk test but not the Brown-Forsythe test, p-values are from Welch's 1-way ANOVA with Dunnett's T3 multiple comparisons test (repression by EsaR); for data that did not pass the Shapiro-Wilk or Brown-Forsythe tests, p-values are from Kruskal-Wallis test with Dunn's multiple comparisons test (repression by LE-2 or LE-4, and activation by EL-3, LX-1, or LX-2).

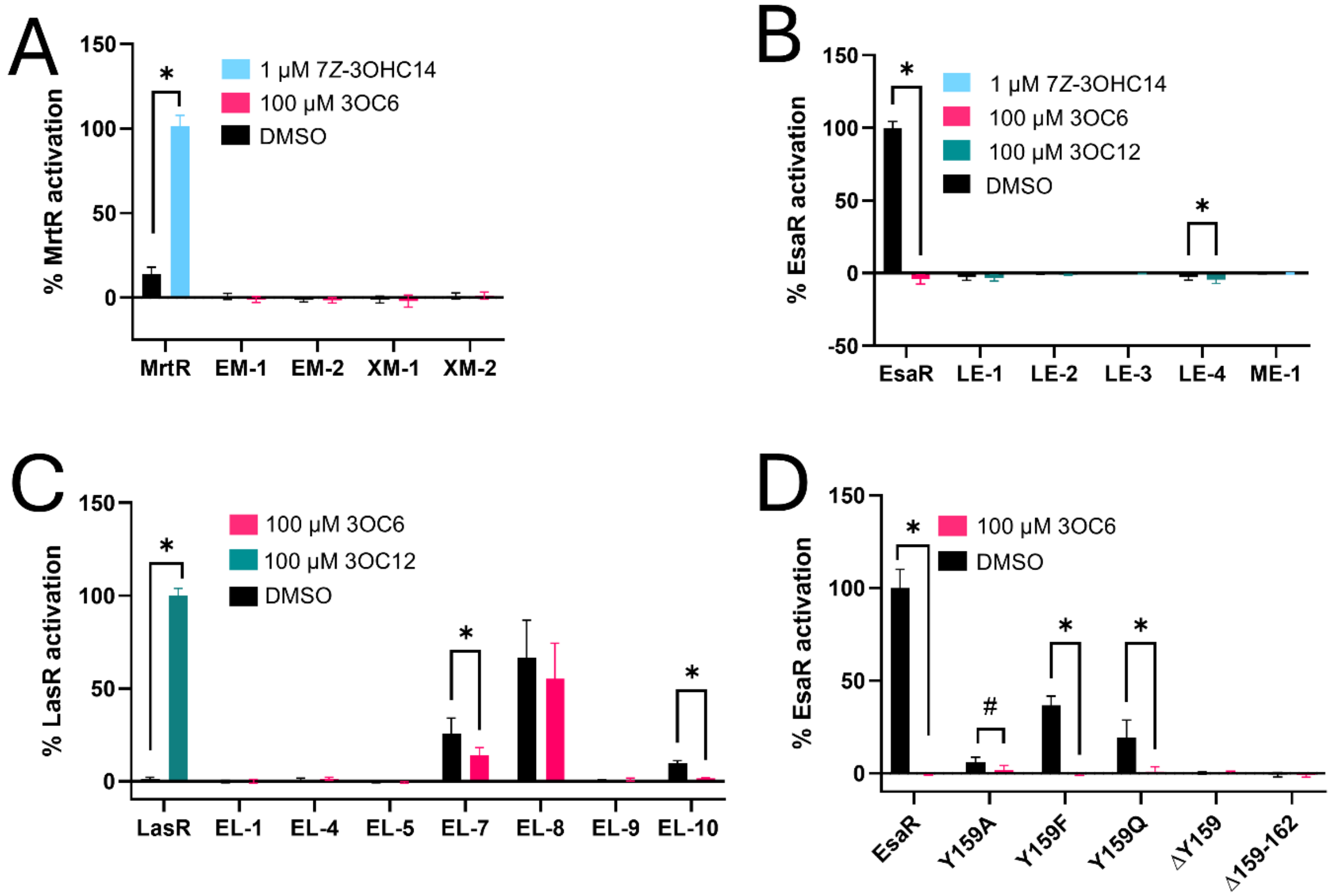

Figure S4. Additional chimera and mutant activity data. **(A)** Transcriptional activation of the *mrtI* promoter by chimeras with an MrtR DBD in an *E. coli* JLD271 reporter, normalized to uninduced culture (0%) and WT MrtR saturated with 7Z-3OHC14 (100%). Data represent at least four biological replicates, each performed in technical triplicate. WT MrtR activity is graphed for reference. **(B)** Transcriptional activation of the *esaS* promoter by chimeras with an EsaR DBD in an *E. coli* JLD271 reporter, normalized to uninduced culture (0%) and maximum EsaR activation (100%). Data represent at least two biological replicates, each performed in technical triplicate. WT EsaR activity is graphed for reference. **(C)** Transcriptional activation of the *lasI* promoter by chimeras with a LasR DBD (those not shown in **Figure 4B**) in a JLD271 reporter, normalized to uninduced culture (0%) and LasR saturated with 3OC12 (100%). Data represent at least three biological replicates, each performed in technical triplicate. WT LasR activity is graphed for reference. **(D)** Transcriptional activation of the *esaS* promoter by EsaR mutants in a BW27749 reporter, normalized to uninduced culture (0%) and maximum EsaR activation (100%). Data represent at least three biological replicates, each performed in technical triplicate. WT EsaR activity is graphed for reference. **(All graphs)** Error bars indicate standard deviation. Asterisks and hash marks indicate that activity is different with and without ligand with $p \leq 0.01$ or $p \leq 0.05$, respectively, based on multiple Mann-Whitney tests with the Holm-Šídák correction for multiple comparisons.

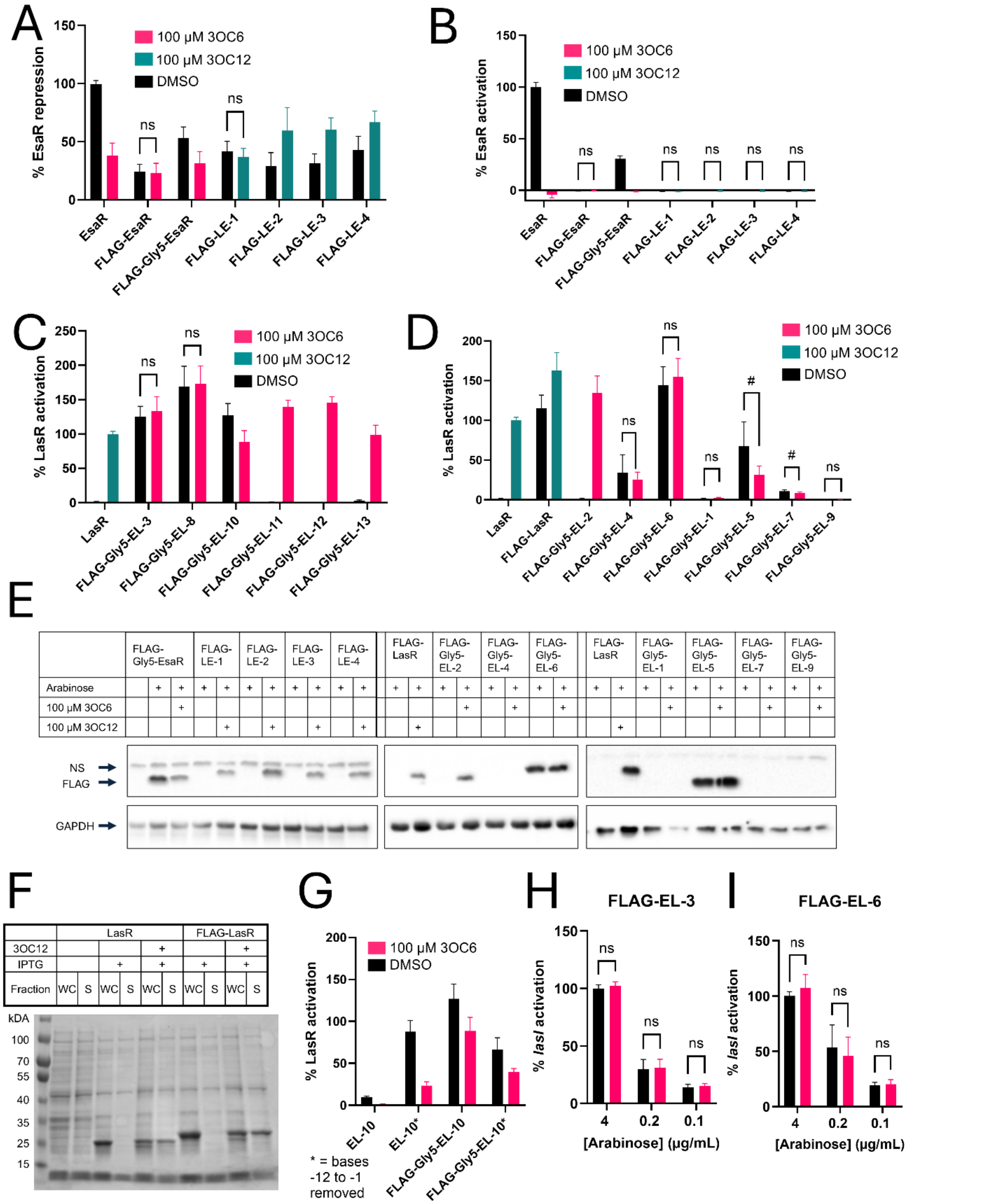

Figure S5. Effects of FLAG tag on activity. (A) Transcriptional repression of the *esaR* promoter by FLAG-tagged constructs with an EsaR DBD in an *E. coli* JLD271 reporter, normalized to uninduced culture (0%) and maximum EsaR repression (100%). Data represent at least two biological replicates, each performed in technical triplicate. WT EsaR activity is graphed for reference. **(B)** Transcriptional activation of the *esaS* promoter by FLAG-tagged constructs with an EsaR DBD in a JLD271 reporter,

normalized to uninduced culture (0%) and maximum EsaR activation (100%). Data represent at least two biological replicates, each performed in technical triplicate. WT EsaR activity is graphed for reference. **(C)** and **(D)** Transcriptional activation of the *lasI* promoter by FLAG-tagged constructs with a LasR DBD in a JLD271 reporter, normalized to uninduced culture (0%) and LasR saturated with 3OC12 (100%). Data represent at least three biological replicates, each performed in technical duplicate or triplicate. WT LasR activity is graphed for reference. **(E)** Western blots of whole cell lysate from reporter strains used to collect data shown in panels **(B)** and **(D)**. Images are representative of two biological replicates. See **Figure S12A** for additional replicates and end of this SI document for full blot images. Note that nonspecific binding to GFP, the production of which is induced by FLAG-Gly5-EsaR in the absence of ligand, could affect the intensity of the FLAG-Gly5-EsaR bands. **(F)** Solubility test of LasR and FLAG-LasR overexpressed in *E. coli* BL21(DE3) in the presence and absence of 10 μM 3OC12. WC represents whole cell lysate and S represents soluble fraction. **(G)** Transcriptional activation of the *lasI* promoter in a JLD271 reporter by chimera EL-10 with and without a FLAG tag and/or 12 base pairs removed just upstream of the start codon, normalized to uninduced culture (0%) and LasR saturated with 3OC12 (100%). Data represent at least three biological replicates, each performed in technical triplicate. Data for EL-10 and FLAG-Gly5-EL10 are also shown in **Figure S4C** and **S5C**, respectively. Compound key applies to panels **G**, **H,** and **I**. **(H)** Transcriptional activation of the *lasI* promoter by FLAG-EL-3 in the presence of different concentrations of arabinose, normalized to FLAG-EL-3 activation with 4 μg/mL arabinose and no compound (DMSO) (100%). Data represent two biological replicates, each performed in technical triplicate. Activity was not significantly different with and without ligand even with a cutoff of $p \leq 0.1$. **(I)** Transcriptional activation of the *lasI* promoter by FLAG-EL-6 in the presence of different concentrations of arabinose, normalized to FLAG-EL-6 activation with 4 μg/mL arabinose and no compound (DMSO) (100%). Data represent two biological replicates, each performed in technical triplicate. Activity was not significantly different with and without ligand even with a cutoff of $p \leq 0.1$. **(All graphs)** Error bars indicate standard deviation. Activity was significantly different with and without ligand based on multiple Mann-Whitney U tests with the Holm-Šídák correction for multiple comparisons and $p \leq 0.01$ unless indicated with a hash mark ($0.01 \leq p \leq 0.05$) or as ns (not significant).

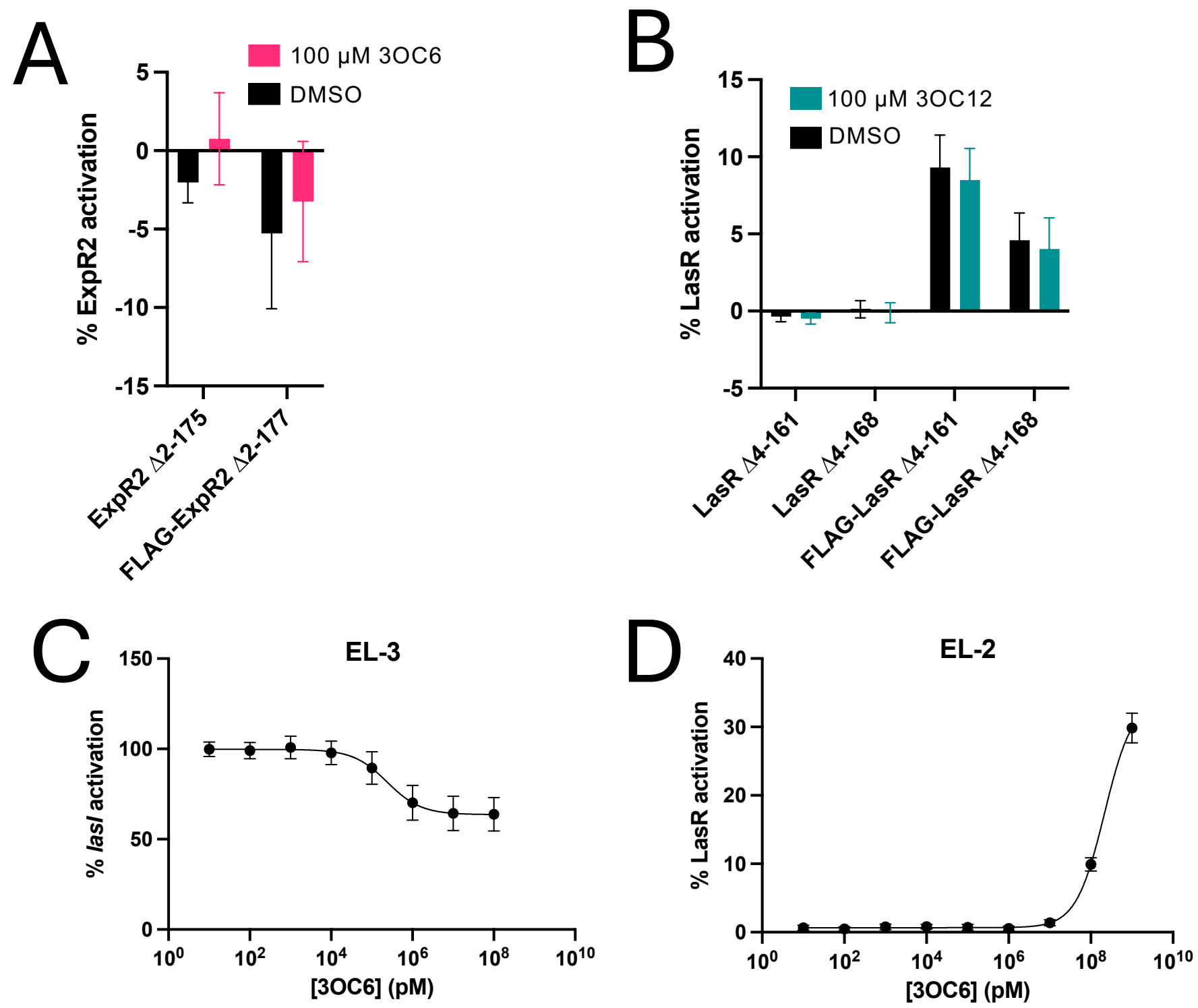

Figure S6. Activity of DBDs alone. (A) Transcriptional activation of the *rsmA* promoter by ExpR2 truncations in an *E. coli* JLD271 reporter, normalized to uninduced culture (0%) and maximum ExpR2 activation (100%). **(B)** Transcriptional activation of the *lasI* promoter by LasR truncations in a JLD271 reporter, normalized to uninduced culture (0%) and LasR saturated with 3OC12 (100%).
(C) Transcriptional activation of the *lasI* promoter by chimera EL-3, normalized to uninduced culture (0%) and mutant activity with no compound (DMSO) (100%). **(D)** Transcriptional activation of the *lasI* promoter by chimera EL-2, normalized to uninduced culture (0%) and LasR saturated with 3OC12 (100%). **(All graphs)** Data represent at least three biological replicates, each performed in technical triplicate. Error bars indicate standard deviation.

A

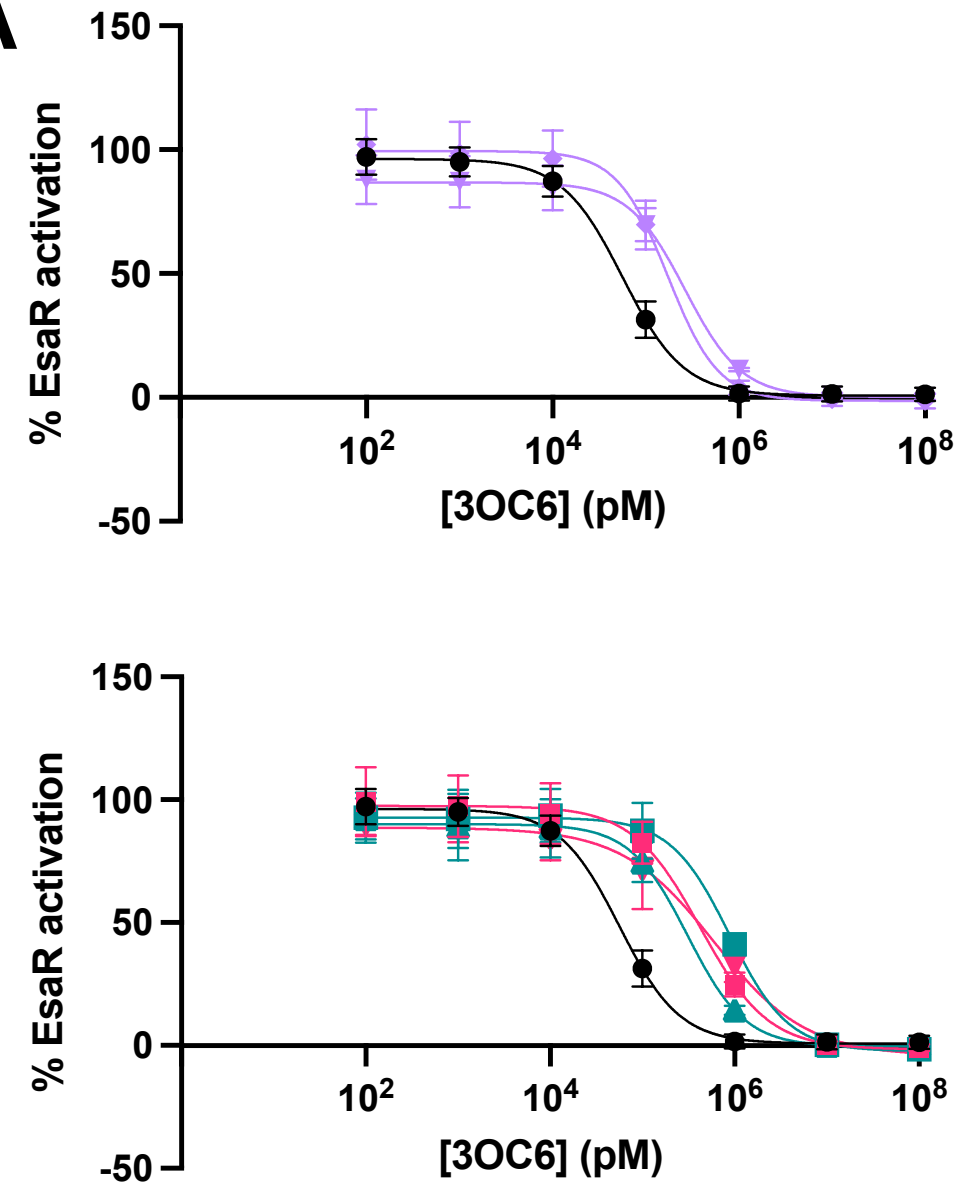

Construct	EC_{50} (95% CI) (nM)
EsaR	56 (49-64)
Δ4: 168-171 (RAPA)	260 (200-340)
Δ4: 172-175 (LNQS)	170 (140-210)
Δ6: 164-169 (TEGERA)	880 (710-1100)
Δ6: 171-176 (ALNQSA)	310 (240-400)
Δ8: 164-171 (TEGERAPA)	420 (330-540)
Δ8: 168-169, 171-176 (RA, ALNQSA)	600 (430-820)

B

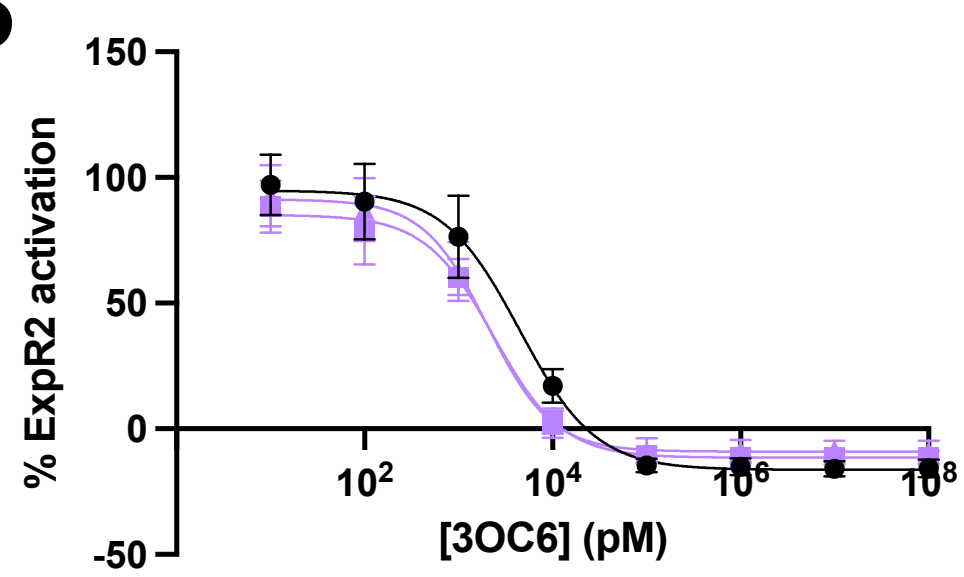

Construct	EC_{50} (95% CI) (nM)
ExpR2	4.5 (3.4-5.8)
Δ169-172 (PQSR)	2.4 (2.0-2.8)
Δ173-176 (KQGD)	2.0 (1.7-2.5)

Figure S7. Ligand potency in EsaR and ExpR2 linker deletion mutants. (A) Dose-response curves and EC_{50} values for EsaR linker mutants. Graphs show transcriptional activation of the *esaS* promoter in an *E. coli* BW27749 reporter, normalized to uninduced culture (0%) and maximum EsaR activation (100%). WT EsaR activity is plotted on both graphs for comparison. **(B)** Dose-response curves and EC_{50} values for ExpR2 linker mutants showing transcriptional activation of the *rsmA* promoter in an *E. coli* JLD271 reporter, normalized to uninduced culture (0%) and maximum ExpR2 activation (100%). WT ExpR2 activity is plotted for comparison. **(All graphs)** Data represent at least three biological replicates, each performed in technical triplicate. Error bars indicate standard deviation. CI = confidence interval. Some data from dose-response curves are also included in on/off plots in **Figure 5**.

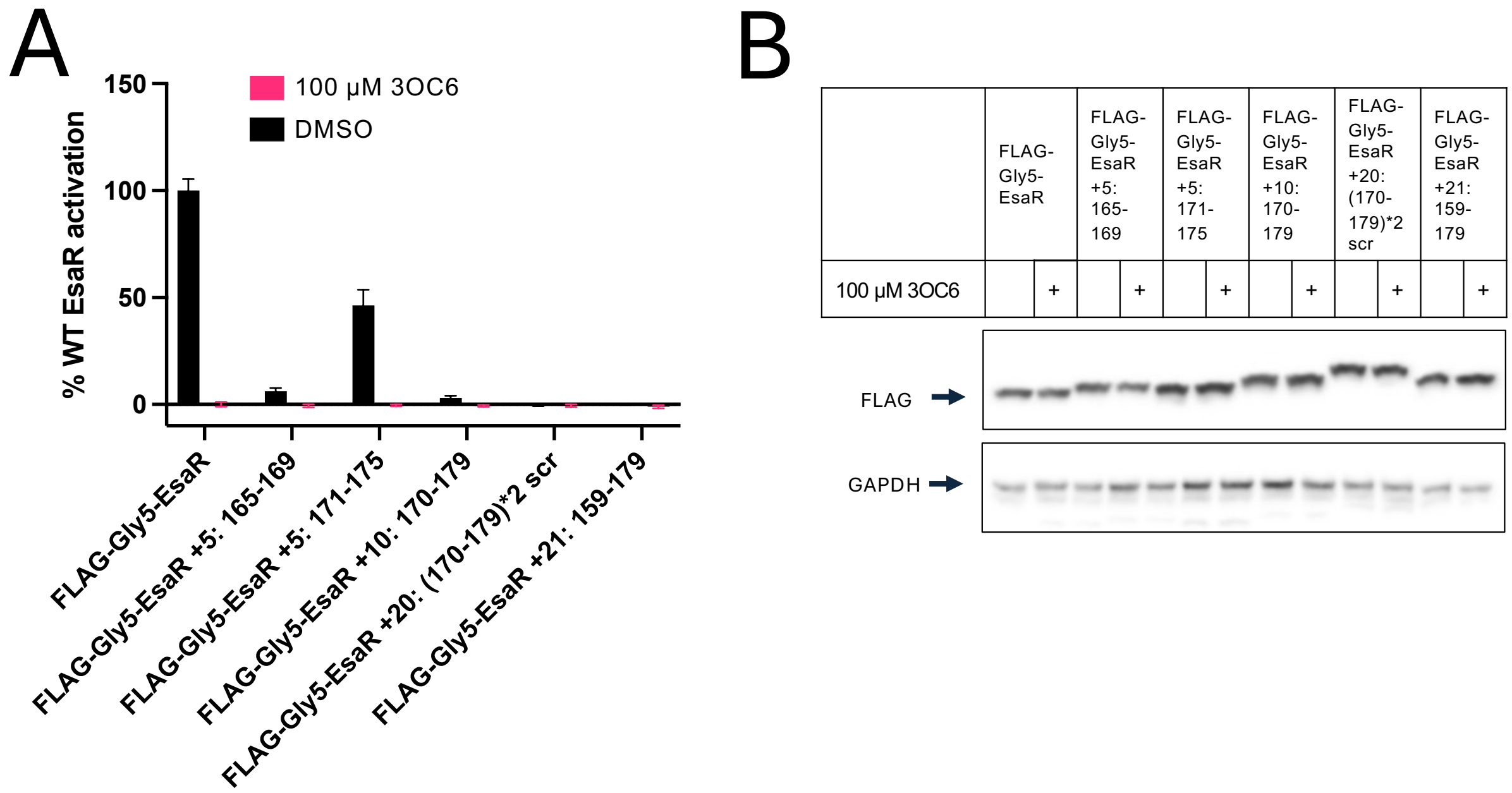

Figure S8. Activity and accumulation of FLAG-tagged EsaR linker addition mutants.
(A) Transcriptional activation of the *esaS* promoter in an *E. coli* BW27749 reporter induced with 0.4 mg/mL arabinose, normalized to uninduced culture (0%) and FLAG-Gly5-EsaR activation with DMSO (100%). Data represent at least three biological replicates, each performed in technical triplicate. Error bars indicate standard deviation. **(B)** Western blots of whole cell lysate from reporter strains used to collect data shown in panel **A**. Images are representative of two biological replicates. Additional replicate is shown in **Figure S12B**, and full blot images are shown at the end of this SI document.

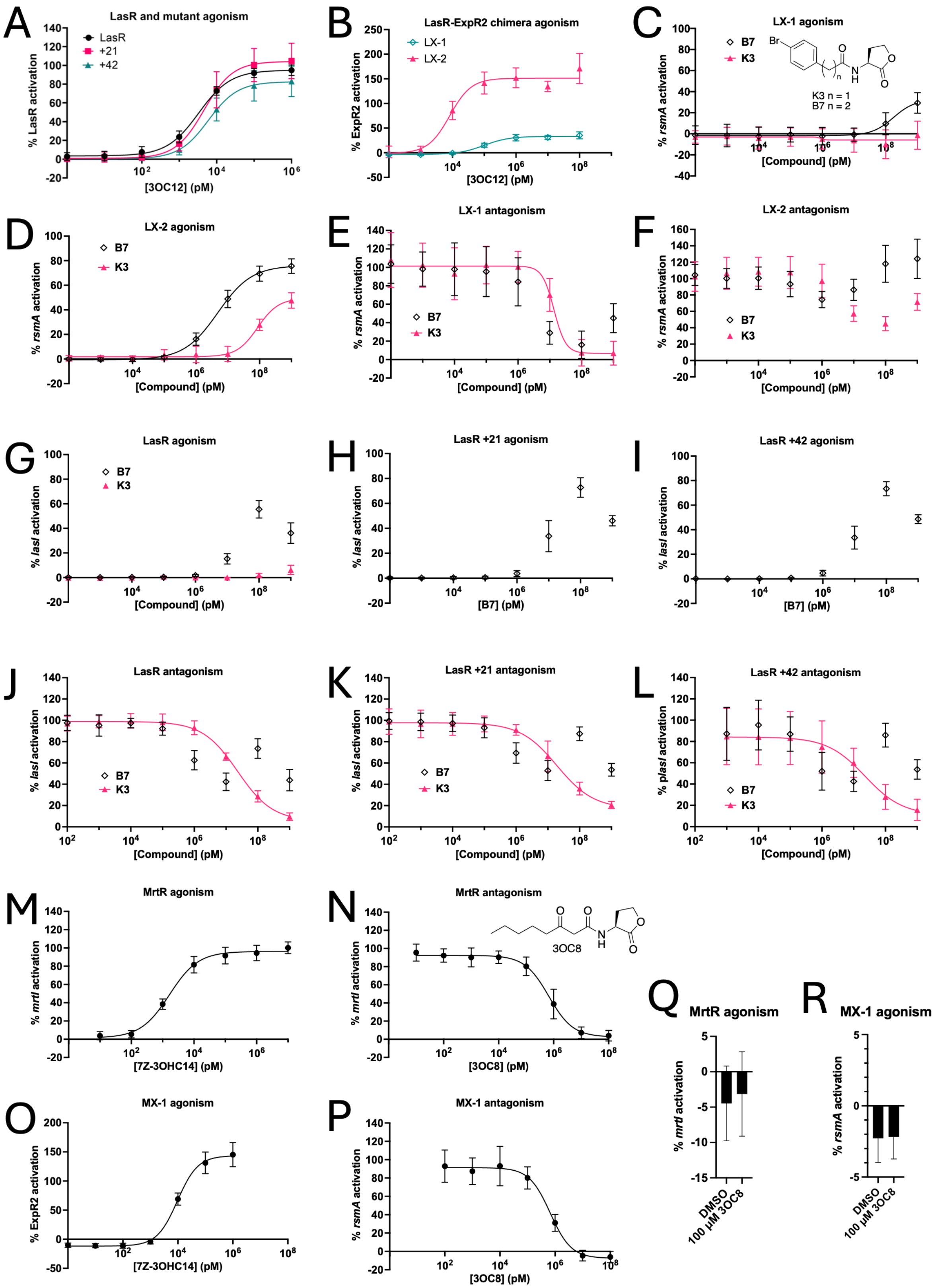
A
LasR and mutant agonism
LasR
+21
+42
% LasR activation
[3OC12] (pM)
B
LasR-ExpR2 chimera agonism
LX-1
LX-2
% ExpR2 activation
[3OC12] (pM)
C
LX-1 agonism
B7
K3
Br
K3 n = 1
B7 n = 2
% rsmA activation
[Compound] (pM)
D
LX-2 agonism
% rsmA activation
[Compound] (pM)
E
LX-1 antagonism
% rsmA activation
[Compound] (pM)
F
LX-2 antagonism
% rsmA activation
[Compound] (pM)
G
LasR agonism
% lasI activation
[Compound] (pM)
H
LasR +21 agonism
% lasI activation
[B7] (pM)
I
LasR +42 agonism
% lasI activation
[B7] (pM)
J
LasR antagonism
% lasI activation
[Compound] (pM)
K
LasR +21 antagonism
% lasI activation
[Compound] (pM)
L
LasR +42 antagonism
% plasI activation
[Compound] (pM)
M
MrtR agonism
% mrtI activation
[7Z-3OHC14] (pM)
N
MrtR antagonism
3OC8
% mrtI activation
[3OC8] (pM)
O
MX-1 agonism
% ExpR2 activation
[7Z-3OHC14] (pM)
P
MX-1 antagonism
% rsmA activation
[3OC8] (pM)
Q
MrtR agonism
% mrtI activation
DMSO
100 µM 3OC8
R
MX-1 agonism
% rsmA activation
DMSO
100 µM 3OC8

Figure S9. Small molecule inhibition of select mutants and chimeras (*figure on previous page)***.** **(A)** Transcriptional activation of the *lasI* promoter by LasR linker extensions in an *E. coli* BW27749 reporter, normalized to uninduced culture (0%) and LasR saturated with 3OC12 (100%). See **Table S2** for sequence details. **(B)** Transcriptional activation of the *rsmA* promoter by chimeras with an ExpR2 DBD in an *E. coli* JLD271 reporter, normalized to uninduced culture (0%) and maximum ExpR2 activation (100%). Data represent at least two biological replicates, each performed in technical triplicate. **(C)** and **(D)** Transcriptional activation of the *rsmA* promoter by the LasR-ExpR2 chimeras LX-1 **(C)** and LX-2 **(D)** in a JLD271 reporter, normalized to DMSO (0%) and each mutant saturated with 3OC12 (100%). **(E)** Transcriptional activation of the *rsmA* promoter by chimera LX-1 in a JLD271 reporter, normalized to chimera LX-1 with DMSO (0%) and 100 nM 3OC12 (100%). Compounds were competed against 100 nM 3OC12. **(F)** Transcriptional activation of the *rsmA* promoter by LX-2 in a JLD271 reporter, normalized to LX-2 with DMSO (0%) and 10 nM 3OC12 (100%). Compounds were competed against 10 nM 3OC12. **(G-I)** Transcriptional activation of the *lasI* promoter by LasR **(G)**, LasR +21 **(H)**, and LasR +42 **(I)** in a BW27749 reporter, normalized to each construct with DMSO (0%) and saturated with 3OC12 (100%). **(J-L)** Transcriptional activation of the *lasI* promoter by LasR **(J)**, LasR +21 **(K)**, and LasR +42 **(L)** in a BW27749 reporter, normalized to each construct with DMSO (0%) and 10 nM 3OC12 (100%). Compounds were competed against 10 nM 3OC12. **B7** data collected in technical duplicate or triplicate. **(M)** and **(Q)** Transcriptional activation of the *mrtI* promoter by MrtR in a BW27749 reporter, normalized to uninduced culture (0%) and MrtR saturated with 7*Z*-3OHC14 (100%). **(N)** Transcriptional activation of the *mrtI* promoter by MrtR in a BW27749 reporter, normalized to uninduced culture (0%) and MrtR with 8 nM 7*Z*-3OHC14 (100%). Compound was competed against 8 nM 7*Z*-3OHC14. **(O)** Transcriptional activation of the *rsmA* promoter by MrtR-ExpR2 chimera MX-1 in a JLD271 reporter, normalized to uninduced culture (0%) and maximum ExpR2 activation (100%). **(P)** Transcriptional activation of the *rsmA* promoter by chimera MX-1 in a JLD271 reporter, normalized to uninduced culture (0%) and MX-1 with 40 nM 7*Z*-3OHC14 (100%). Compound was competed against 40 nM 7*Z*-3OHC14. **(R)** Transcriptional activation of the *rsmA* promoter by chimera MX-1 in a JLD271 reporter, normalized to uninduced culture (0%) and MX-1 with 1 μM 7*Z*-3OHC14 (100%). **(All graphs)** Unless indicated, data represent at least three biological replicates, each performed in technical triplicate. Error bars indicate standard deviation. Some data from panels **A** and **B** are also shown in **Figures 5** and **4**, respectively.

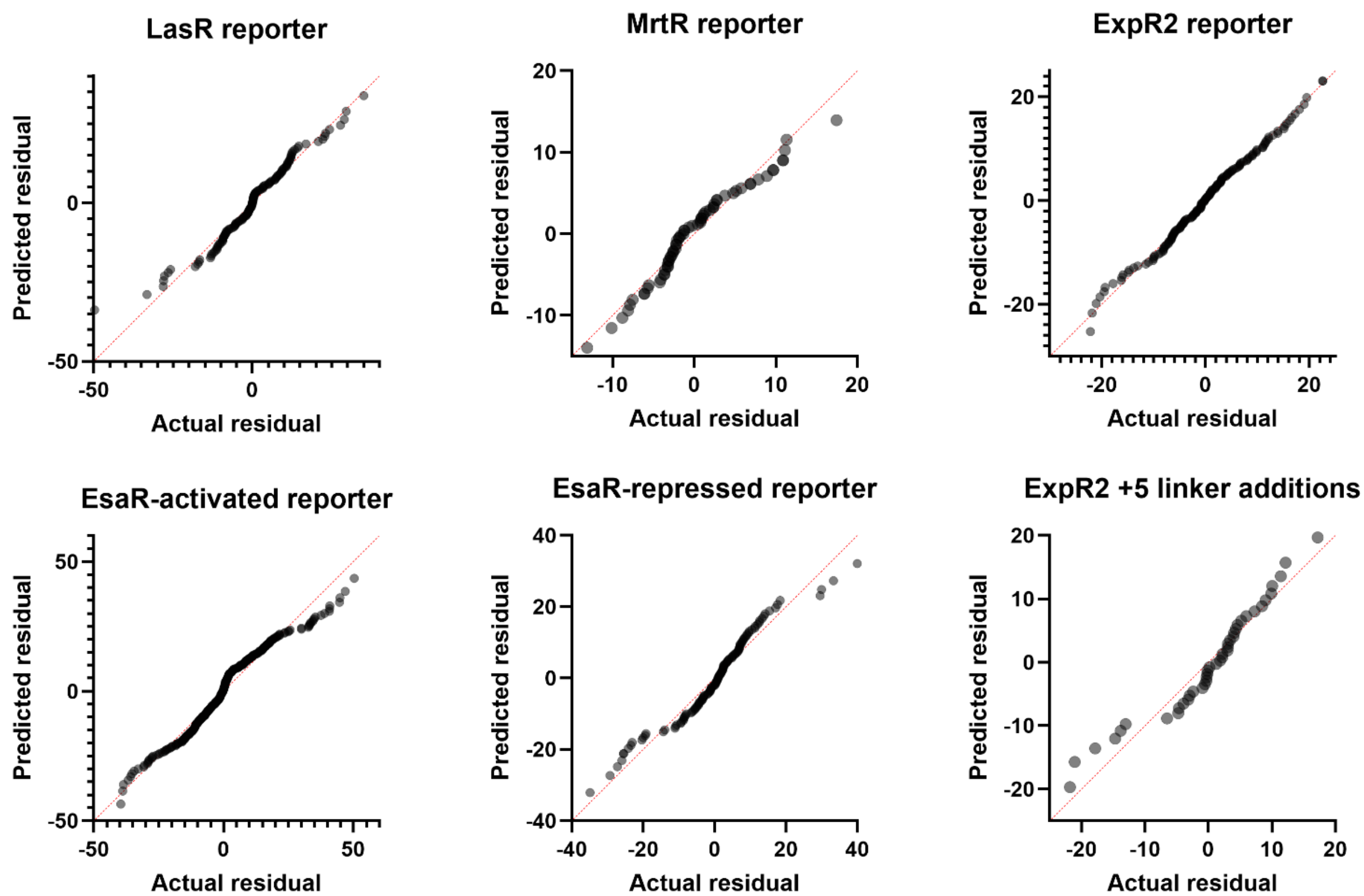

Figure S10. Quantile-Quantile plots. The ExpR2 +5 linker additions plot corresponds to all ExpR2 mutants analyzed in **Table S4**, and the other plots correspond to data shown in **Figure 5.**

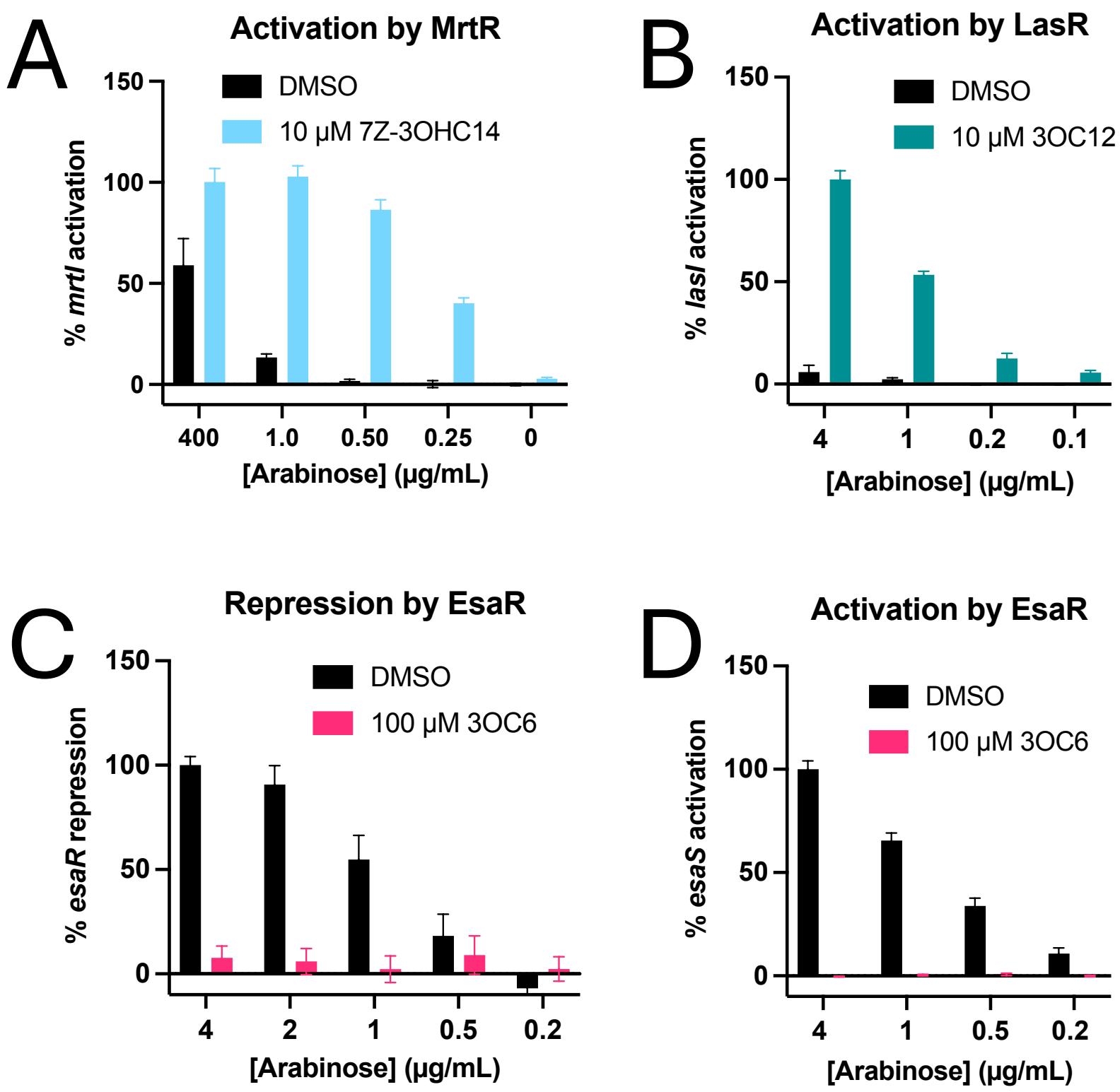

Figure S11. Optimization of arabinose concentration for _E. coli_ BW27749 reporters.
(A) Transcriptional activation of the _mrtI_ promoter by MrtR in a BW27749 reporter, normalized to uninduced culture (0%) and 400 µg/mL arabinose with 7Z-3OHC14 (100%). Data represent at least two biological replicates, each performed in technical triplicate. **(B)** Transcriptional activation of the _lasI_ promoter by LasR in a BW27749 reporter, normalized to uninduced culture (0%) and 4 µg/mL arabinose with 3OC12 (100%). Data represent two biological replicates, each performed in technical triplicate. **(C)** Transcriptional repression of the _esaR_ promoter by EsaR in a BW27749 reporter, normalized to uninduced culture (0%) and 4 µg/mL arabinose with DMSO (100%). Data represent three biological replicates, each performed in technical triplicate. **(D)** Transcriptional activation of the _esaS_ promoter by EsaR in a BW27749 reporter, normalized to uninduced culture (0%) and 4 µg/mL arabinose with DMSO (100%). Data represent at least two biological replicates, each performed in technical triplicate. **(All graphs)** Error bars indicate standard deviation.

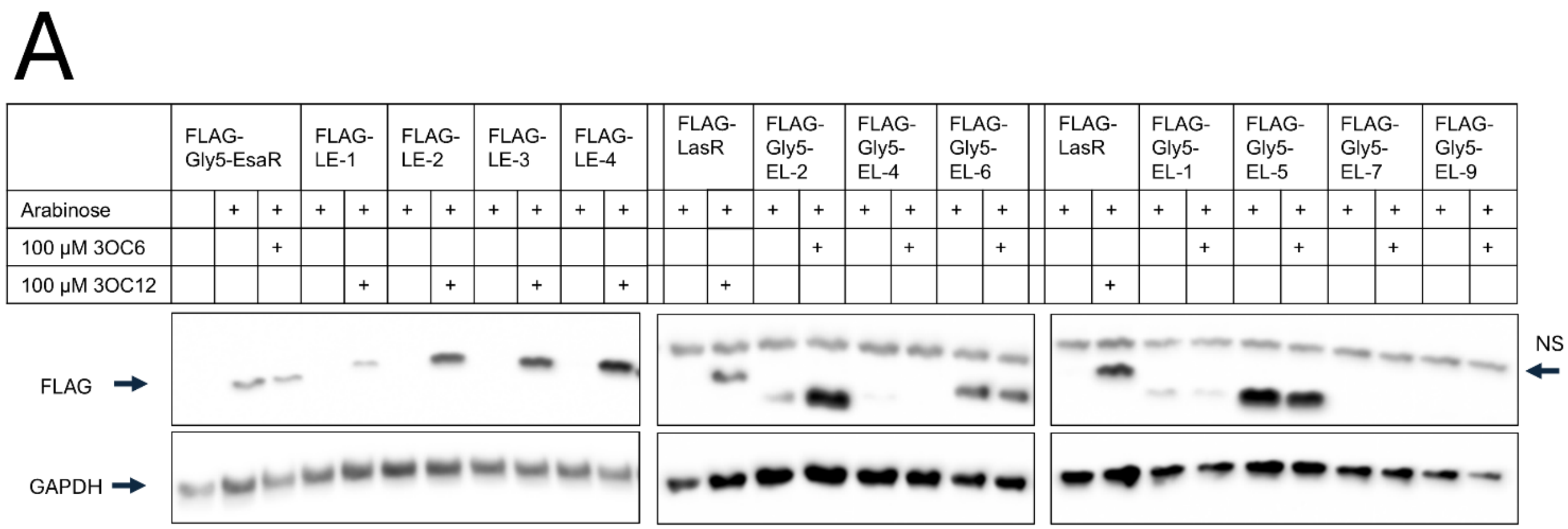

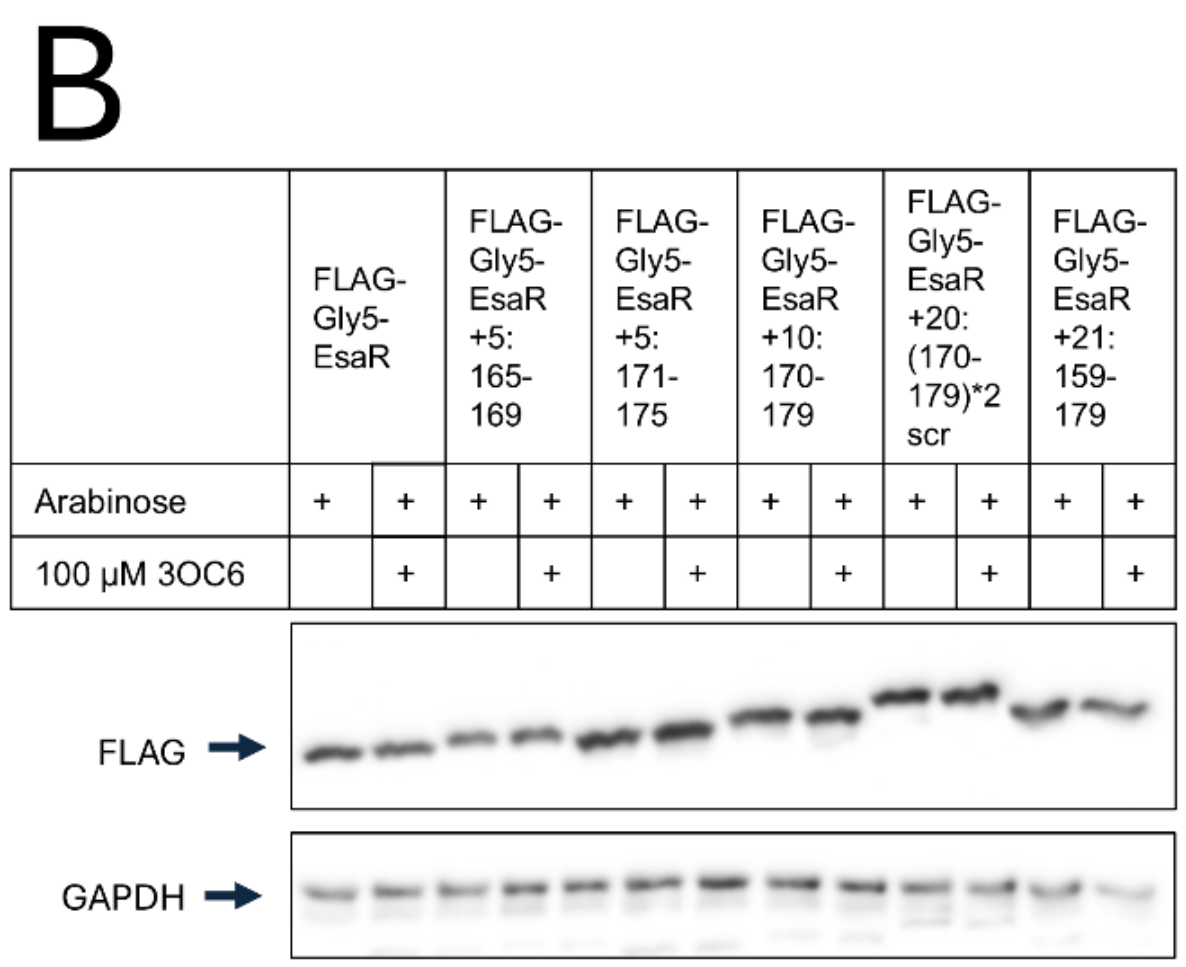

Figure S12. Additional biological replicate of western blots in **Figure S5E (A)** and **Figure S8B (B)**. "NS" indicates nonspecific band observed in FLAG blot.

Table S1. Protein sequences of WT LuxR-type receptors examined in this study.

Protein	Sequence
**LasR**	MALVDGFLELERSSGKLEWSAILQKMASDLGFSKILFGLLPKDSQDYENAFIVGNYPAAWRE<br>HYDRAGYARVDPTVSHCTQSVLPIFWEPSIYQTRKQHEFFEEASAAGLVYGLTMPLHGARG<br>ELGALSLSVEAENRAEANRFMESVLPTLWMLKDYALQSGAGLAFEHPVSKPVVLTSREKEV<br>LQWCAIGKTSWEISVICNCSEANVNFHMGNIRRKFGVTSRRVAAIMAVNLGLITL
**EsaR**	MFSFFLENQTITDTLQTYIQRKLSPLGSPDYAYTVVSKKNPSNVLIISSYPDEWIRLYRANNFQ<br>LTDPVILTAFKRTSPFAWDENITLMSDLRFTKIFSLSKQYNIVNGFTYVLHDHMNNLALLSVIIK<br>GNDQTALEQRLAAEQGTMQMLLIDFNEQMYRLAGTEGERAPALNQSADKTIFSSRENEVLY<br>WASMGKTYAEIAAITGISVSTVKFHIKNVVVKLGVSNARQAIRLGVELDLIRPAASAAR
**ExpR2**	MSVFCSDNEIINNTIKSYLGRKLKQYGDLKYAYMIMNKKNPSQVVIISNYPQEWVNTYKENN<br>YQHIDPVILTAINKVSPFSWEDNIVINSKLKFSKIFNLSKEYDIVNGYTFVLHDNNNNLAALSIM<br>FEEHAPTDMENIVEENKDKLQMLLIAVHEKITTLYKEMTQSPQSRKQGDKEIFSQRENEILYW<br>ASMGKTYPEIALILDIKISTVKFHIGNVVKKLGVLNAKHAIRLGVELQLIKPEPL
**MrtR**	MIENTYSEKFESAFEQIKAAANVDAAIRILQAEYGLDFVTYHLAQTIAAKIDSPFVRTTYPDAW<br>VSRYLLNSYVKVDPIVKQGFERQLPFDWSEVEPTPEAYAMLVDAQKHGIGGNGYSIPVADK<br>AQRRALLSLNARIPAEEWAELVRRCRNEWIEIAHLIHRKAVYELHGENDPVPALSPREIECLH<br>WTALGKDYKDISVILGISEHTTRDYLKTARFKLGCATISAAASRAVQLRIINP

Table S2. Bacterial strains and plasmids used in this study. Ap^R = ampicillin resistance, Gm^R = gentamycin resistance, Cm^R = chloramphenicol resistance.

**Strain**	**Description**	**Reference**
E. coli Top 10	Used for cloning.	Lab collection
E. coli JLD271	K12 *ΔsdiA*$_{ec}$, used for reporters.	[15]
E. coli BW27749	Constitutive expression of low-affinity arabinose pump AraE and deletion of *araFGH*. Used for arabinose-titratable reporters.	[16]
E. coli BL21(DE3) pLysS	Used for protein overexpression. pLysS confers Cm^R.	Novagen
**Plasmid**	**Description**	**Reference**
pJN105L	Arabinose-inducible lasR expression vector; pBBRMCS backbone (Gm^R)	[10]
pSC11L	Broad host range p*lasI*-lacZ reporter (Ap^R)	[17]
pJN105-esaR	pJN105 with arabinose-inducible promoter controlling *esaR* (Gm^R)	[18]
pesaR-AC:GFP	p*esaS* promoter from *Pantoea stewartii* subsp. *stewartii* controlling GFP (Ap^R)	[7]
pesaR:GFP	p*esaR* promoter from *P. stewartii* controlling *GFP* (Ap^R)	[18]
pJN105-expR2	pJN105 with arabinose-inducible promoter controlling *expR2* (Gm^R)	[18]
prsmA:GFP	*rsmA* promoter from *Pectobacterium versatile* Ecc71 controlling *GFP* (Ap^R)	[18]
pJN105-mrtR	pJN105 with arabinose-inducible promoter controlling *mrtR* (Gm^R)	This study
pmrtI:GFP	*mrtI* promoter (-120 to -1 relative the *mrtI* translational start site) from *Mesorhizobium tianshanense* controlling *GFP* (Ap^R)	This study
pET17b-lasR	Vector for LasR overexpression in BL21(DE3) (Ap^R)	[19]
pET17b-FLAG-lasR[a]	pET17b-lasR with a FLAG tag inserted between residues 1 and 2 (Ap^R)	This study
pJN105-EL-1	pJN105L with LasR residues 1-151 replaced with EsaR 1-147 (Gm^R)	This study
pJN105-EL-2	pJN105L with LasR residues 1-161 replaced with EsaR 1-158 (Gm^R)	This study
pJN105-EL-3	pJN105L with LasR residues 1-168 replaced with EsaR 1-168 (Gm^R)	This study
pJN105-EL-4	pJN105L with LasR residues 1-177 replaced with EsaR 1-181 (Gm^R)	This study
pJN105-EL-5	pJN105L with LasR residues 1-168 replaced with EsaR 1-163 (Gm^R)	This study

pJN105-EL-6	pJN105L with LasR residues 1-168 replaced with EsaR 1-179 (Gm^R)	This study
pJN105-EL-7	pJN105L with LasR residues 1-161 replaced with EsaR 1-162 (Gm^R)	This study
pJN105-EL-8	pJN105L with LasR residues 1-161 replaced with EsaR 1-168 (Gm^R)	This study
pJN105-EL-9	pJN105L with LasR residues 1-135 replaced with EsaR 1-131 (Gm^R)	This study
pJN105-EL-10	pJN105L with LasR residues 1-172 replaced with EsaR 1-168 (Gm^R)	This study
pJN105-EL-10*	pJN105-EL-10 with base pairs -12 to -1 removed (Gm^R)	This study
pJN105-EL-11	pJN105L with LasR residues 1-157 replaced with EsaR 1-158 (Gm^R)	This study
pJN105-EL-12	pJN105L with LasR residues 1-160 replaced with EsaR 1-158 (Gm^R)	This study
pJN105-EL-13	pJN105L with LasR residues 1-165 replaced with EsaR 1-158 (Gm^R)	This study
pJN105-XL-1	pJN105L with LasR residues 1-161 replaced with ExpR2 1-158 (Gm^R)	This study
pJN105-XL-2	pJN105L with LasR residues 1-172 replaced with ExpR2 1-168 (Gm^R)	This study
pJN105-LE-1	pJN105-esaR with EsaR residues 1-147 replaced with LasR 1-151 (Gm^R)	This study
pJN105-LE-2	pJN105-esaR with EsaR residues 1-158 replaced with LasR 1-161 (Gm^R)	This study
pJN105-LE-3	pJN105-esaR with EsaR residues 1-168 replaced with LasR 1-168 (Gm^R)	This study
pJN105-LE-4	pJN105-esaR with EsaR residues 1-181 replaced with LasR 1-177 (Gm^R)	This study
pJN105-ME-1	pJN105-esaR with EsaR residues 1-172 replaced with MrtR 1-174 (Gm^R)	This study
pJN105-LX-1	pJN105-expR2 with ExpR2 residues 1-158 replaced with LasR 1-161 (Gm^R)	This study
pJN105-LX-2	pJN105-expR2 with ExpR2 residues 1-168 replaced with LasR 1-172 (Gm^R)	This study
pJN105-MX-1	pJN105-expR2 with ExpR2 residues 1-169 replaced with MrtR 1-174 (Gm^R)	This study
pJN105-XM-1	pJN105-mrtR with MrtR residues 1-165 replaced with ExpR2 1-158 (Gm^R)	This study
pJN105-XM-2	pJN105-mrtR with MrtR residues 1-174 replaced with ExpR2 1-170 (Gm^R)	This study

pJN105-EM-1	pJN105-mrtR with MrtR residues 1-174 replaced with EsaR 1-168 (Gm^R)	This study
pJN105-EM-2	pJN105-mrtR with MrtR residues 1-170 replaced with EsaR 1-168 (Gm^R)	This study
pJN105-FLAG-LasR[a]	pJN105L with a FLAG tag inserted between residues 1 and 2 (Gm^R)	This study
pJN105-FLAG-Gly5-EL1[b]	pJN105-EL-1 with a FLAG-Gly5 tag inserted between residues 1 and 2 (Gm^R)	This study
pJN105-FLAG-Gly5-EL2[b]	pJN105-EL-2 with a FLAG-Gly5 tag inserted between residues 1 and 2 (Gm^R)	This study
pJN105-FLAG-Gly5-EL3[b]	pJN105-EL-3 with a FLAG-Gly5 tag inserted between residues 1 and 2 (Gm^R)	This study
pJN105-FLAG-Gly5-EL4[b]	pJN105-EL-4 with a FLAG-Gly5 tag inserted between residues 1 and 2 (Gm^R)	This study
pJN105-FLAG-Gly5-EL5[b]	pJN105-EL-5 with a FLAG-Gly5 tag inserted between residues 1 and 2 (Gm^R)	This study
pJN105-FLAG-Gly5-EL6[b]	pJN105-EL-6 with a FLAG-Gly5 tag inserted between residues 1 and 2 (Gm^R)	This study
pJN105-FLAG-Gly5-EL7[b]	pJN105-EL-7 with a FLAG-Gly5 tag inserted between residues 1 and 2 (Gm^R)	This study
pJN105-FLAG-Gly5-EL8[b]	pJN105-EL-8 with a FLAG-Gly5 tag inserted between residues 1 and 2 (Gm^R)	This study
pJN105-FLAG-Gly5-EL9[b]	pJN105-EL-9 with a FLAG-Gly5 tag inserted between residues 1 and 2 (Gm^R)	This study
pJN105-FLAG-Gly5-EL10[b]	pJN105-EL-10 with a FLAG-Gly5 tag inserted between residues 1 and 2 (Gm^R)	This study
pJN105-FLAG-Gly5-EL10*[b]	pJN105-FLAG-Gly5-EL10 with base pairs -12 to -1 deleted (Gm^R)	This study
pJN105-FLAG-Gly5-EL11[b]	pJN105-EL-11 with a FLAG-Gly5 tag inserted between residues 1 and 2 (Gm^R)	This study
pJN105-FLAG-Gly5-EL12[b]	pJN105-EL-12 with a FLAG-Gly5 tag inserted between residues 1 and 2 (Gm^R)	This study
pJN105-FLAG-Gly5-EL13[b]	pJN105-EL-13 with a FLAG-Gly5 tag inserted between residues 1 and 2 (Gm^R)	This study
pJN105-lasR +21	pJN105L with EsaR residues 159-179 inserted after LasR 168 (Gm^R)	This study
pJN105-lasR +42	pJN105L with EsaR residues 159-179 doubled and inserted after LasR 168 (Gm^R)	This study
pJN105-lasR +105	pJN105L with an insertion after LasR 168 of EsaR residues 159-168, (EsaR 159-179)*4 scrambled,[c] EsaR 169-179 (Gm^R)	This study
pJN105-lasR ΔP170	pJN105L with a Δ170 mutation (Gm^R)	This study
pJN105-lasR ΔV171	pJN105L with a Δ171 mutation (Gm^R)	This study

pJN105-lasR ΔS172	pJN105L with a Δ172 mutation (Gm^R)	This study
pJN105-lasR ΔK173	pJN105L with a Δ173 mutation (Gm^R)	This study
pJN105-lasR Δ169, 171	pJN105L with Δ169 and Δ171 mutations (Gm^R)	This study
pJN105-lasR Δ172-173	pJN105L with a Δ172-173 mutation (Gm^R)	This study
pJN105-lasR Δ169, 171-172	pJN105L with Δ169 and Δ171-172 mutations (Gm^R)	This study
pJN105-lasR Δ170-172	pJN105L with a Δ170-172 mutation (Gm^R)	This study
pJN105-lasR Scr 169-174	pJN105L with residues H169-P174 scrambled (HPVSKP to SPKPVH) (Gm^R)	This study
pJN105-lasR Δ4-161	pJN105-lasR with residues 4 to 161 deleted (Gm^R)	This study
pJN105-lasR Δ4-168	pJN105-lasR with residues 4 to 168 deleted (Gm^R)	This study
pJN105-FLAG-lasR Δ4-161[a]	pJN105-lasR Δ4-161 with a FLAG tag inserted between residues 3 and 4 of the mutant (Gm^R)	This study
pJN105-FLAG-lasR Δ4-168[a]	pJN105-lasR Δ4-168 with a FLAG tag inserted between residues 3 and 4 of the mutant (Gm^R)	This study
pJN105-Myc-lasR	pJN105-lasR with a Myc tag (EQKLISEEDL) inserted between residues 1 and 2 (Gm^R)	This study
pJN105-His6-lasR	pJN105-lasR with 6 His residues (HHHHHH) inserted between residues 1 and 2 (Gm^R)	This study
pJN105-HA-lasR	pJN105-lasR with an HA tag (YPYDVPDYA) inserted between residues 1 and 2 (Gm^R)	This study
pJN105-Gly6-lasR	pJN105-lasR with 6 Gly residues (GGGGGG) inserted between residues 1 and 2 (Gm^R)	This study
pJN105-NLS-lasR	pJN105-lasR with an NLS tag (PKKKRKV) inserted between residues 1 and 2 (Gm^R)	This study
pJN105-lasR Δ2-10	pJN105L with residues 2-10 deleted (Gm^R)	This study
pJN105-FLAG-esaR[a]	pJN105-esaR with a FLAG tag inserted between residues 1 and 2 (Gm^R)	This study
pJN105-FLAG-Gly5-esaR[b]	pJN105-esaR with a FLAG-Gly5 tag inserted between residues 1 and 2 (Gm^R)	This study
pJN105-FLAG-LE-1[a]	pJN105-LE-1 with a FLAG tag inserted between residues 1 and 2 (Gm^R)	This study
pJN105-FLAG-LE-2[a]	pJN105-LE-2 with a FLAG tag inserted between residues 1 and 2 (Gm^R)	This study
pJN105-FLAG-LE-3[a]	pJN105-LE-3 with a FLAG tag inserted between residues 1 and 2 (Gm^R)	This study
pJN105-FLAG-LE-4[a]	pJN105-LE-4 with a FLAG tag inserted between residues 1 and 2 (Gm^R)	This study
pJN105-esaR +5: 165-169	pJN105-esaR with residues 165-169 (EGERA) doubled (Gm^R)	This study
pJN105-esaR +5: 171-175	pJN105-esaR with residues 171-175 (ALNQS) doubled (Gm^R)	This study

pJN105-esaR +5G at 171	pJN105-esaR with 5 glycine residues inserted after residue 171 (Gm^R)	This study
pJN105-esaR +5G at 176	pJN105-esaR with 5 glycine residues inserted after residue 176 (Gm^R)	This study
pJN105-esaR +5A at 171	pJN105-esaR with 5 alanine residues inserted after residue 171 (Gm^R)	This study
pJN105-esaR +5A at 176	pJN105-esaR with 5 alanine residues inserted after residue 176 (Gm^R)	This study
pJN105-esaR +10: 170-179	pJN105-esaR with residues 170-179 (PALNQSADKT) doubled (Gm^R)	This study
pJN105-esaR +20: (170-179)*2 scr	pJN105-esaR with ALSTQPSKDNLPNQAATKDA (Residues 170-179 (PALNQSADKT) doubled and scrambled) inserted after residue 169 (Gm^R)	This study
pJN105-esaR +21: 159-179	pJN105-esaR with residues 159-179 (YRLAGTEGERAPALNQSADKT) doubled (Gm^R)	This study
pJN105-esaR Δ164-167	pJN105-esaR with residues 164-167 (TEGE) deleted (Gm^R)	This study
pJN105-esaR Δ168-171	pJN105-esaR with residues 168-171 (RAPA) deleted (Gm^R)	This study
pJN105-esaR Δ172-175	pJN105-esaR with residues 172-175 (LNQS) deleted (Gm^R)	This study
pJN105-esaR Δ176-179	pJN105-esaR with residues 176-179 (ADKT) deleted (Gm^R)	This study
pJN105-esaR Δ164-169	pJN105-esaR with residues 164-169 (TEGERA) deleted (Gm^R)	This study
pJN105-esaR Δ171-176	pJN105-esaR with residues 171-176 (ALNQSA) deleted (Gm^R)	This study
pJN105-esaR Δ168-169, 171-176	pJN105-esaR with residues 168-169 (RA) and 171-176 (ALNQSA) deleted (Gm^R)	This study
pJN105-esaR Δ164-171	pJN105-esaR with residues 164-171 (TEGERAPA) deleted (Gm^R)	This study
pJN105-esaR Δ164-175	pJN105-esaR with residues 164-175 (TEGERAPALNQS) deleted (Gm^R)	This study
pJN105-esaR Scr 172-177	pJN105-esaR with residues 172-177 (LNQSAD) scrambled to DQLASN (Gm^R)	This study
pJN105-esaR ΔY159	pJN105-esaR with a deletion of Y159 (Gm^R)	This study
pJN105-esaR Δ159-162	pJN105-esaR with a deletion of residues 159-162 (Gm^R)	This study
pJN105-esaR Y159A	pJN105-esaR with a Y159A mutation (Gm^R)	This study
pJN105-esaR Y159F	pJN105-esaR with a Y159F mutation (Gm^R)	This study
pJN105-esaR Y159Q	pJN105-esaR with a Y159F mutation (Gm^R)	This study
pJN105-esaR G163A	pJN105-esaR with a G163A mutation (Gm^R)	This study

pJN105-esaR T164A	pJN105-esaR with a T164A mutation (Gm^R)	This study
pJN105-esaR E165A	pJN105-esaR with a E165A mutation (Gm^R)	This study
pJN105-esaR G166A	pJN105-esaR with a G166A mutation (Gm^R)	This study
pJN105-esaR E167A	pJN105-esaR with a E167A mutation (Gm^R)	This study
pJN105-esaR R168A	pJN105-esaR with a R168A mutation (Gm^R)	This study
pJN105-esaR P170A	pJN105-esaR with a P170A mutation (Gm^R)	This study
pJN105-esaR L172A	pJN105-esaR with a L172A mutation (Gm^R)	This study
pJN105-esaR N173A	pJN105-esaR with a N173A mutation (Gm^R)	This study
pJN105-esaR Q174A	pJN105-esaR with a Q174A mutation (Gm^R)	This study
pJN105-esaR S175A	pJN105-esaR with a S175A mutation (Gm^R)	This study
pJN105-esaR D177A	pJN105-esaR with a D177A mutation (Gm^R)	This study
pJN105-esaR K178A	pJN105-esaR with a K178A mutation (Gm^R)	This study
pJN105-esaR T179A	pJN105-esaR with a T179A mutation (Gm^R)	This study
pJN105-esaR I180A	pJN105-esaR with a I180AA mutation (Gm^R)	This study
pJN105-FLAG-Gly5-esaR +5: 165-169[b]	pJN105-esaR +5: 165-169 with a FLAG-Gly5 tag inserted between residues 1 and 2 (Gm^R)	This study
pJN105-FLAG-Gly5-esaR +5: 171-175[b]	pJN105-esaR +5: 171-175 with a FLAG-Gly5 tag inserted between residues 1 and 2 (Gm^R)	This study
pJN105-FLAG-Gly5-esaR +10: 170-179[b]	pJN105-esaR +10: 170-179 with a FLAG-Gly5 tag inserted between residues 1 and 2 (Gm^R)	This study
pJN105-FLAG-Gly5-esaR +20: (170-179)*2 scr[b]	pJN105-esaR +20: (170-179)*2 scr with a FLAG-Gly5 tag inserted between residues 1 and 2 (Gm^R)	This study
pJN105-FLAG-Gly5-esaR +21: 159-179[b]	pJN105-esaR +21: 159-179 with a FLAG-Gly5 tag inserted between residues 1 and 2 (Gm^R)	This study
pJN105-esaR F4A	pJN105-esaR with an F4A mutation (Gm^R)	This study
pJN105-esaR F5A	pJN105-esaR with an F5A mutation (Gm^R)	This study
pJN105-mrtR +4G at 171	pJN105-mrtR with 4 glycine residues inserted after residue 171 (Gm^R)	This study
pJN105-mrtR +4G at 175	pJN105-mrtR with 4 glycine residues inserted after residue 175 (Gm^R)	This study
pJN105-mrtR +4A at 171	pJN105-mrtR with 4 alanine residues inserted after residue 171 (Gm^R)	This study
pJN105-mrtR +4A at 175	pJN105-mrtR with 4 alanine residues inserted after residue 175 (Gm^R)	This study
pJN105-mrtR +5A at 171	pJN105-mrtR with 5 alanine residues inserted after residue 171 (Gm^R)	This study
pJN105-mrtR +5A at 175	pJN105-mrtR with 5 alanine residues inserted after residue 175 (Gm^R)	This study
pJN105-mrtR Δ171-173	pJN105-mrtR with residues 171-173 (GEN) deleted (Gm^R)	This study

pJN105-expR2 +5G at 171	pJN105-expR2 with 5 glycine residues inserted after residue 171 (Gm^R)	This study
pJN105-expR2 +5G at 176	pJN105-expR2 with 5 glycine residues inserted after residue 176 (Gm^R)	This study
pJN105-expR2 +5A at 171	pJN105-expR2 with 5 alanine residues inserted after residue 171 (Gm^R)	This study
pJN105-expR2 +5A at 176	pJN105-expR2 with 5 alanine residues inserted after residue 176 (Gm^R)	This study
pJN105-expR2 +5: 172-176	pJN105-expR2 with residues 172-176 (RKQGD) doubled (Gm^R)	This study
pJN105-expR2 +10: 168-177	pJN105-expR2 with residues 168-177 (SPQSRKQGDK) doubled (Gm^R)	This study
pJN105-expR2 +21:158-178	pJN105-expR2 with residues 158-178 (ITTLYKEMTQSPQSRKQGDKE) doubled (Gm^R)	This study
pJN105-expR2 Δ169-172	pJN105-expR2 with residues 169-172 (PQSR) deleted (Gm^R)	This study
pJN105-expR2 Δ173-176	pJN105-expR2 with residues 173-176 (KQGD) deleted (Gm^R)	This study
pJN105-expR2 Δ170-175	pJN105-expR2 with residues 170-175 (QSRKQG) deleted (Gm^R)	This study
pJN105-expR2 Δ169-176	pJN105-expR2 with residues 169-176 (PQSRKQGD) deleted (Gm^R)	This study
pJN105-expR2 K177A	pJN105-expR2 with a K177A mutation (Gm^R)	This study
pJN105-expR2 E178A	pJN105-expR2 with an E178A mutation (Gm^R)	This study
pJN105-expR2 F4A	pJN105-expR2 with an F4A mutation (Gm^R)	This study
pJN105-expR2 Δ2-175	pJN105-expR2 with residues 2-175 deleted (Gm^R)	This study
pJN105-FLAG-expR2 Δ2-177[a]	pJN105-expR2 with residues 2-177 deleted and a FLAG tag added between residues 1 and 2 of the mutant[d] (Gm^R)	This study

[a] Sequence of FLAG tag: GATTATAAAGATGATGATGATAAA.

[b] Sequence of FLAG-Gly5 tag: GATTATAAAGATGATGATGATAAAGGTGGTGGTGGTGGC.

[c] Amino acid sequence of (EsaR 159-179)*4 scrambled that was inserted to make LasR +105 (see **Table S6** for gBlock): PYTTEARDRNLADLTGETNLKQASYATRGESGEYQRAKDAKGAGAARAAPGQPLALA RNPLEATELKYESLARTATRDNSQGEG.

[d] Analogous to ExpR2 Δ2-175 because the last 2 residues of the FLAG tag replace native D176 and K177.

Table S3. Ordinary one-way ANOVA with Šídák's multiple comparisons test for activation by EsaR +5 linker mutants with DMSO, corresponding to data in **Figure 5F**. Data passed the Brown-Forsythe test for homogeneity of variance and the Shapiro-Wilk test for normality.

Comparison	Mean diff.	99.00% CI of diff.	Adjusted p value
**+5A at 171 vs. +5G at 171**	-21	-36 to -5	<0.001
**+5A at 171 vs. +5A at 176**	-23	-39 to -8	<0.001
**+5G at 171 vs. +5G at 176**	-13	-28 to 2	0.038
**+5A at 176 vs. +5G at 176**	-10	-26 to 5	0.134

Table S4. Ordinary one-way ANOVA with Šídák's multiple comparisons test for activation by ExpR2 +5 linker mutants with DMSO, corresponding to data in **Figure 5G**. Data passed the Brown-Forsythe test for homogeneity of variance, and the quantile-quantile plot showed only minor deviations from normality (**Figure S10**).

Comparison	Mean diff.	99.00% CI of diff.	Adjusted p value
**+5A at 171 vs. +5G at 171**	-28	-41 to -16	<0.001
**+5A at 171 vs. +5A at 176**	30	17 to 42	<0.001
**+5G at 171 vs. +5G at 176**	44	31 to 58	<0.001
**+5A at 176 vs. +5G at 176**	-14	-27 to -0.2	0.008

Table S5. Ordinary one-way ANOVA with Šídák's multiple comparisons test for activation by MrtR +4 linker mutants with 7Z-3OHC14, corresponding to data in **Figure 5B**. Data passed the Brown-Forsythe test for homogeneity of variance and the Shapiro-Wilk test for normality.

Comparison	Mean diff.	99.00% CI of diff.	Adjusted p value
**+4G at 171 vs. +4A at 171**	-5	-15 to 6	0.474
**+4G at 171 vs. +4G at 175**	23	12 to 33	<0.001
**+4A at 171 vs. +4A at 175**	27	17 to 37	<0.001
**+4G at 175 vs. +4A at 175**	-0.4	-11 to 10	0.999

Table S6. gBlocks used in this study.

gBlock	Sequence
mrtI promoter fragment used for pmrtI:GFP	CTGCACACTTTTGCGCGATATGCGCCCCCCTCATCTGAGGGGGCCCATCTGAGG<br>GAATTTCCGAACCGGCCCGCTTGAACCATTCTGCTTTCCACGAACTTGAAAACGC<br>TGGAGGGCAAA
Codon-optimized *mrtR* sequence	ATGATCGAAAACACATATTCGGAGAAGTTCGAGAGTGCGTTCGAGCAAATCAAAG<br>CTGCTGCGAATGTCGATGCCGCAATTCGTATCCTTCAGGCAGAATATGGGTTGGA<br>TTTCGTCACGTATCACTTGGCGCAAACCATCGCCGCCAAAATCGACTCGCCATTC<br>GTTCGCACTACATATCCTGATGCGTGGGTCAGCCGCTACCTGCTGAACAGCTATG<br>TCAAGGTGGACCCAATCGTGAAGCAAGGATTTGAACGCCAATTGCCTTTTGATTG<br>GAGTGAAGTGGAGCCCACACCTGAGGCGTACGCCATGCTGGTCGATGCACAGAA<br>ACACGGAATCGGAGGTAACGGATACTCTATTCCAGTTGCCGACAAGGCCCAACG<br>TCGCGCATTACTGTCGTTAAACGCTCGTATCCCTGCCGAAGAGTGGGCGGAGTT<br>GGTACGTCGTTGTCGCAACGAATGGATTGAGATTGCACATTTGATTCATCGCAAA<br>GCGGTTTACGAGTTGCACGGGGAGAACGATCCGGTCCCCGCCCTTTCGCCTCGT<br>GAAATCGAATGCCTGCATTGGACTGCCCTTGGTAAGGATTACAAGGATATCTCAG<br>TGATTTTAGGTATCAGTGAGCACACCACGCGTGATTACCTGAAAACTGCCCGTTTT<br>AAATTAGGTTGCGCGACCATCTCAGCAGCGGCCAGCCGTGCCGTTCAGCTTCGC<br>ATCATCAATCCGTAA
gBlock used to create pJN105-lasR +105	GTGCCGGACTGGCCTTCGAATACCGCCTGGCAGGCACCGAAGGTGAACGACCTT<br>ACACTACGGAAGCTCGTGACCGTAACTTAGCAGACCTGACAGGTGAAACAAACCT<br>TAAGCAGGCTTCCTATGCTACCCGTGGAGAATCTGGGGAGTATCAGCGCGCAAA<br>AGACGCCAAGGGGGCAGGCGCGGCGCGTGCTGCGCCGGGCCAACCTCTTGCC<br>CTGGCTCGTAACCCTTTGGAAGCGACTGAATTAAAGTATGAGTCCCTTGCTCGTA<br>CAGCGACACGTGACAATAGCCAGGGTGAAGGTGCCCCGGCGTTAAATCAGAGCG<br>CGGACAAAACGCATCCGGTCAGCAAACCGGTG

Table S7. Primers used in this study.

Plasmid	Primer name	Sequence
pJN105-EL-1	Vector (from pJN105L) FWD	CTGCCGAACAGGGCACGATGTGGATGCTCAAGGACTACGC
	Vector REV	TCAAGGAAGAAAGAGAACATAGCGCTACGTTCTTCTTAAGA
	Insert (from pJN105-esaR) FWD	CTTAAGAAGAACGTAGCGCTATGTTCTCTTTCTTCCTTGAAAA
	Insert REV	GCGTAGTCCTTGAGCATCCACATCGTGCCCTGTTCGGCAG
pJN105-LE-1	Vector (from pJN105-esaR) FWD	AGTCGGTCCTGCCGACCCTGCAGATGCTGCTGATTGATTT
	Vector REV	AAACCGTCAACCAAGGCCATGAATTCGCTAGCCCAAAAAAACG
	Insert (from pET17b-lasR) FWD	TTTTTTGGGCTAGCGAATTCATGGCCTTGGTTGACGGTTT
	Insert REV	AAATCAATCAGCAGCATCTGCAGGGTCGGCAGGACCGACT
pJN105-EL-2	Vector (from pJN105L) FWD	TTGATTTTAACGAGCAGATGGGTGCCGGACTGGCCTTCGA
	Vector REV	TCAAGGAAGAAAGAGAACATAGCGCTACGTTCTTCTTAAGA
	Insert (from pJN105-esaR) FWD	CTTAAGAAGAACGTAGCGCTATGTTCTCTTTCTTCCTTGAAAA
	Insert REV	TCGAAGGCCAGTCCGGCACCCATCTGCTCGTTAAAATCAATC
pJN105-LE-2	Vector (from pJN105-esaR) FWD	AGGACTACGCACTGCAGAGCTACCGCCTGGCAGGCACCGA
	Vector REV	AAACCGTCAACCAAGGCCATGAATTCGCTAGCCCAAAAAAACG
	Insert (from pET17b-lasR) FWD	TTTTTTGGGCTAGCGAATTCATGGCCTTGGTTGACGGTTT
	Insert REV	TCGGTGCCTGCCAGGCGGTAGCTCTGCAGTGCGTAGTCCT
pJN105-EL-3	Vector (from pJN105L) FWD	CAGGCACCGAAGGTGAACGACATCCGGTCAGCAAACCGGT
	Vector REV	TCAAGGAAGAAAGAGAACATAGCGCTACGTTCTTCTTAAG
	Insert (from pJN105-esaR) FWD	CTTAAGAAGAACGTAGCGCTATGTTCTCTTTCTTCCTTGAAAA
	Insert REV	ACCGGTTTGCTGACCGGATGTCGTTCACCTTCGGTGCCTG

pJN105-LE-3	Vector (from pJN105-esaR) FWD	GTGCCGGACTGGCCTTCGAAGCCCCGGCGTTAAATCAGAG
	Vector REV	AAACCGTCAACCAAGGCCATGAATTCGCTAGCCCAAAAAA
	Insert (from pET17b-lasR) FWD	TTTTTTGGGCTAGCGAATTCATGGCCTTGGTTGACGGTTT
	Insert REV	CTCTGATTTAACGCCGGGGCTTCGAAGGCCAGTCCGGCAC
pJN105-EL-4	Vector (from pJN105L) FWD	GCGCGGACAAAACGATATTTACCAGCCGGGAGAAGGAAGT
	Vector REV	TCAAGGAAGAAAGAGAACATAGCGCTACGTTCTTCTTAAGA
	Insert (from pJN105-esaR) FWD	CTTAAGAAGAACGTAGCGCTATGTTCTCTTTCTTCCTTGAAAA
	Insert REV	ACTTCCTTCTCCCGGCTGGTAAATATCGTTTTGTCCGC
pJN105-LE-4	Vector (from pJN105-esaR) FWD	TCAGCAAACCGGTGGTTCTGTCCTCGCGTGAAAATGAGGT
	Vector REV	AAACCGTCAACCAAGGCCATGAATTCGCTAGCCCAAAAAAACG
	Insert (from pET17b-lasR) FWD	TTTTTTGGGCTAGCGAATTCATGGCCTTGGTTGACGGTTT
	Insert REV	ACCTCATTTTCACGCGAGGACAGAACCACCGGTTTGCTGA
pJN105-EL-5	Vector (from pJN105L) FWD	AGATGTACCGCCTGGCAGGCCATCCGGTCAGCAAACCG
	Vector REV	TCAAGGAAGAAAGAGAACATAGCGCTACGTTCTTCTTAAGA
	Insert (from pJN105-esaR) FWD	CTTAAGAAGAACGTAGCGCTATGTTCTCTTTCTTCCTTGAAAA
	Insert REV	ACCGGTTTGCTGACCGGATGGCCTGCCAGGCGGTACATC
pJN105-EL-6	Vector (from pJN105L) FWD	ATCAGAGCGCGGACAAAACGCATCCGGTCAGCAAACCG
	Vector REV	TCAAGGAAGAAAGAGAACATAGCGCTACGTTCTTCTTAAGA
	Insert (from pJN105-esaR) FWD	CTTAAGAAGAACGTAGCGCTATGTTCTCTTTCTTCCTTGAAAA
	Insert REV	CGGTTTGCTGACCGGATGCGTTTTGTCCGCGCTCTGAT
pJN105-EL-7	Vector (from pJN105L) FWD	AGCAGATGTACCGCCTGGCAGGTGCCGGACTGGCCTTCGA

	Vector REV	TCAAGGAAGAAAGAGAACATAGCGCTACGTTCTTC TTAAGA
	Insert (from pJN105-esaR) FWD	CTTAAGAAGAACGTAGCGCTATGTTCTCTTTCTTC CTTGAAAA
	Insert REV	TCGAAGGCCAGTCCGGCACCTGCCAGGCGGTACA TCTGCT
pJN105-EL-8	Vector (from pJN105L) FWD	CAGGCACCGAAGGTGAACGAGGTGCCGGACTGG CCTTCGA
	Vector REV	TCAAGGAAGAAAGAGAACATAGCGCTACGTTCTTC TTAAGA
	Insert (from pJN105-esaR) FWD	CTTAAGAAGAACGTAGCGCTATGTTCTCTTTCTTC CTTGAAAA
	Insert REV	TCGAAGGCCAGTCCGGCACCTCGTTCACCTTCGG TGCCTG
pJN105-EL-9	Vector (from pJN105L) FWD	CCGTGATCATTAAAGGCAACAACCGGGCCGAGGC CAAC
	Vector REV	TCAAGGAAGAAAGAGAACATAGCGCTACGTTCTTC TTAAGA
	Insert (from pJN105-esaR) FWD	CTTAAGAAGAACGTAGCGCTATGTTCTCTTTCTTC CTTGAAAA
	Insert REV	CGGTTGGCCTCGGCCCGGTTGTTGCCTTTAATGA TCACGG
pJN105-EL-10*	Vector (from pJN105L) FWD	AGGCACCGAAGGTGAACGAAAACCGGTGGTTCTG ACCAG
	Vector REV	TCAAGGAAGAAAGAGAACATTTCTTAAGAATTCGC TAGCCC
	Insert (from pJN105-esaR) FWD	GGCTAGCGAATTCTTAAGAAATGTTCTCTTTCTTCC TTGAAAACCA
	Insert REV	CTGGTCAGAACCACCGGTTTTCGTTCACCTTCGGT GCCTG
pJN105-EL-10	from pJN105-EL10* FWD	AGCGCTATGTTCTCTTTCTTCCTTG
	from pJN105-EL10* REV	ACGTTCTTCTTAAGAATTCGCTAGC
pJN105-EL-11	Vector (from pJN105L) FWD	GATTTTAACGAGCAGATGGCACTGCAGAGCGGTG
	Vector REV	TCAAGGAAGAAAGAGAACATAGCGCTACGTTCTTC TTAAGA
	Insert (from pJN105-esaR) FWD	CTTAAGAAGAACGTAGCGCTATGTTCTCTTTCTTC CTTGAAAA
	Insert REV	GGCACCGCTCTGCAGTGCCATCTGCTCGTTAAAAT CAATC

pJN105-EL-12	Vector (from pJN105L) FWD	GATTTTAACGAGCAGATGAGCGGTGCCGGACTGG
	Vector REV	TCAAGGAAGAAAGAGAACATAGCGCTACGTTCTTC TTAAGA
	Insert (from pJN105-esaR) FWD	CTTAAGAAGAACGTAGCGCTATGTTCTCTTTCTTC CTTGAAAA
	Insert REV	GGCCAGTCCGGCACCGCTCATCTGCTCGTTAAAA TCAATC
pJN105-EL-13	Vector (from pJN105L) FWD	GATTTTAACGAGCAGATGGCCTTCGAACATCCGGT C
	Vector REV	TCAAGGAAGAAAGAGAACATAGCGCTACGTTCTTC TTAAGA
	Insert (from pJN105-esaR) FWD	CTTAAGAAGAACGTAGCGCTATGTTCTCTTTCTTC CTTGAAAA
	Insert REV	GACCGGATGTTCGAAGGCCATCTGCTCGTTAAAAT CAATC
pJN105-XL-1	Vector (from pJN105L) FWD	TTGCCGTTCATGAGAAAATCGGTGCCGGACTGGC CTTCGA
	Vector REV	TCAGAGCAAAATACAGACATAGCGCTACGTTCTTC TTAAGAATTCGC
	Insert (from pJN105-expR2) FWD	CTTAAGAAGAACGTAGCGCTATGTCTGTATTTTGC TCTGAC
	Insert REV	TCGAAGGCCAGTCCGGCACCGATTTTCTCATGAA CGGCAA
pJN105-XL-2	Vector (from pJN105L) FWD	ACAAAGAGATGACGCAAAGCAAACCGGTGGTTCT GACCAG
	Vector REV	TCAGAGCAAAATACAGACATAGCGCTACGTTCTTC TTAAG
	Insert (from pJN105-expR2) FWD	CTTAAGAAGAACGTAGCGCTATGTCTGTATTTTGC TCTGAC
	Insert REV	CTGGTCAGAACCACCGGTTTGCTTTGCGTCATCTC TTTGT
pJN105-LX-1	Vector (from pJN105-expR2) FWD	AGGACTACGCACTGCAGAGCACCACGCTTTACAA AGAGAT
	Vector REV	AAACCGTCAACCAAGGCCATGAATTCGCTAGCCC AAAAAAACG
	Insert (from pJN105L) FWD	TTTTTTGGGCTAGCGAATTCATGGCCTTGGTTGAC GGTTT
	Insert REV	ATCTCTTTGTAAAGCGTGGTGCTCTGCAGTGCGTA GTCCT
pJN105-LX-2	Vector (from pJN105-expR2) FWD	CCTTCGAACATCCGGTCAGCCCGCAGAGCAGAAA ACAAGG

	Vector REV	AAACCGTCAACCAAGGCCATGAATTCGCTAGCCCAAAAAAACG
	Insert (from pJN105L) FWD	TTTTTTGGGCTAGCGAATTCATGGCCTTGGTTGACGGTTT
	Insert REV	CCTTGTTTTCTGCTCTGCGGGCTGACCGGATGTTCGAAGG
pJN105-mrtR	Vector (from pJN105-esaR) FWD	CGCATCATCAATCCGTAATCTAGAGCGGCCGCCACC
	Vector REV	CGAATATGTGTTTTCGATCATGAATTCGCTAGCCCAAAAA
	Insert (from gBlock) FWD	TGGGCTAGCGAATTCATGATCGAAAACACATATTCGGAG
	Insert REV	GGTGGCGGCCGCTCTAGATTACGGATTGATGATGCGAAGC
pmrtI:GFP	Vector (from pesaR-AC:GFP) FWD	AAAACGCTGGAGGGCAAACCAGCACACTGGCGGCC
	Vector REV	TCGCGCAAAAGTGTGCAGAATTCTTGAAGACGAAAGGGCCT
	Insert (from gBlock) FWD	CTTTCGTCTTCAAGAATTCTGCACACTTTTGCGCGA
	Insert REV	CGGCCGCCAGTGTGCTGGTTTGCCCTCCAGCGTTTT
pJN105-ME-1	Vector (from pJN105-esaR) FWD	TTGCACGGGGAGAACGATAATCAGAGCGCGGACAAA
	Vector REV	CGAATATGTGTTTTCGATCATGAATTCGCTAGCCCAAAAA
	Insert (from pJN105-mrtR) FWD	TGGGCTAGCGAATTCATGATCGAAAACACATATTCGGAG
	Insert REV	TTTGTCCGCGCTCTGATTATCGTTCTCCCCGTGCAA
pJN105-EM-1	Vector (from pJN105-esaR) FWD	CGCATCATCAATCCGTAATCTAGAGCGGCCGCCACC
	Vector REV	CGAAAGGGCGGGGACCGGTCGTTCACCTTCGGTGCC
	Insert (from pJN105-mrtR) FWD	GGCACCGAAGGTGAACGACCGGTCCCCGCCCTTTCG
	Insert REV	GGTGGCGGCCGCTCTAGATTACGGATTGATGATGCGAAGC
pJN105-EM-2	Vector (from pJN105-esaR) FWD	CGCATCATCAATCCGTAATCTAGAGCGGCCGCCACC
	Vector REV	GACCGGATCGTTCTCCCCTCGTTCACCTTCGGTGCC

	Insert (from pJN105-mrtR) FWD	GGCACCGAAGGTGAACGAGGGGAGAACGATCCGGTC
	Insert REV	GGTGGCGGCCGCTCTAGATTACGGATTGATGATGCGAAGC
pJN105-MX-1	Vector (from pJN105-expR) FWD	TTGCACGGGGAGAACGATCAGAGCAGAAAACAAGGTG
	Vector REV	CGAATATGTGTTTTCGATCATGAATTCGCTAGCCCAAAAA
	Insert (from pJN105-mrtR) FWD	TGGGCTAGCGAATTCATGATCGAAAACACATATTCGGAG
	Insert REV	ACCTTGTTTTCTGCTCTGATCGTTCTCCCCGTGCAA
pJN105-XM-1	Vector (from pJN105-expR) FWD	CGCATCATCAATCCGTAATCTAGAGCGGCCGCCACC
	Vector REV	CCCGTGCAACTCGTAAACGATTTTCTCATGAACGGCAATGA
	Insert (from pJN105-mrtR) FWD	GCCGTTCATGAGAAAATCGTTTACGAGTTGCACGGG
	Insert REV	GGTGGCGGCCGCTCTAGATTACGGATTGATGATGCGAAGC
pJN105-XM-2	Vector (from pJN105-expR) FWD	CGCATCATCAATCCGTAATCTAGAGCGGCCGCCACC
	Vector REV	CGAAAGGGCGGGGACCGGCTGCGGGCTTTGCGTCATCT
	Insert (from pJN105-mrtR) FWD	ATGACGCAAAGCCCGCAGCCGGTCCCCGCCCTTTCG
	Insert REV	GGTGGCGGCCGCTCTAGATTACGGATTGATGATGCGAAGC
pJN105-FLAG-lasR	From pJN105-lasR FWD	GATGATGATAAAGCCTTGGTTGACGG
	From pJN105-lasR REV	ATCTTTATAATCCATAGCGCTACGTTC
pJN105-FLAG-esaR	From pJN105-esaR FWD	GATGATGATAAATTCTCTTTCTTCCTTGAAAAC
	From pJN105-esaR REV	ATCTTTATAATCCATGAATTCGCTAGCC
pJN105-FLAG-Gly5-esaR: adding tag to WT and mutants	From pJN105-esaR or mutant FWD	ATAAAGGTGGTGGTGGTGGCTTCTCTTTCTTCCTTGAAAAC
	From pJN105-esaR or mutant REV	CATCATCATCTTTATAATCCATGAATTCGCTAGCC
	From pJN105-EL FWD	ATAAAGGTGGTGGTGGTGGCTTCTCTTTCTTCCTTGAAAAC

Adding FLAG-Gly5 tag to all pJN105-EL chimeras	From pJN105-EL REV	CATCATCATCTTTATAATCCATAGCGCTACGTTC
Adding FLAG tag to pJN105-LE chimeras	From pJN105-LE FWD	GATGATGATAAAGCCTTGGTTGACGG
	From pJN105-LE REV	ATCTTTATAATCCATGAATTCGCTAGCC
pJN105-FLAG-Gly5-EL-10*	From pJN105-EL-10* FWD	ATAAAGGTGGTGGTGGTGGCTTCTCTTTCTTCCTTGAAAAC
	From pJN105-EL-10* REV	CATCATCATCTTTATAATCCATTTCTTAAGAATTCGCTAG
pJN105-lasR +21	Vector (from pJN105-EL-6) FWD	GTGCCGGACTGGCCTTCGAATACCGCCTGGCAGGCACCGA
	Vector REV	AAACCGTCAACCAAGGCCATAGCGCTACGTTCTTCTTAAGAATTCGCT
	Insert (from pET17b-lasR) FWD	CTTAAGAAGAACGTAGCGCTATGGCCTTGGTTGACGGTTT
	Insert REV	TCGGTGCCTGCCAGGCGGTATTCGAAGGCCAGTCCGGCAC
pJN105-lasR +42	Vector (from pJN105-lasR +21) FWD	TGGGTCTTATTACTCTCTGATCTTGCCTCTAGAGCGGCCG
	Vector REV	TCGGTGCCTGCCAGGCGGTACGTTTTGTCCGCGCTCTGAT
	Insert (from pJN105-lasR +21) FWD	ATCAGAGCGCGGACAAAACGTACCGCCTGGCAGGCACCGA
	Insert REV	CGGCCGCTCTAGAGGCAAGATCAGAGAGTAATAAGACCCAAATTAACGGCC
pJN105-lasR +105	Vector (from pJN105L) FWD	CATCCGGTCAGCAAACCGGTG
	Vector REV	TTCGAAGGCCAGTCCGGCAC
	Insert (from gBlock) FWD	GTGCCGGACTGGCCTTCGAA
	Insert REV	CATCCGGTCAGCAAACCGGTG
pJN105-lasR ΔP170	From pJN105L FWD	GTCAGCAAACCGGTGG
	From pJN105L REV	ATGTTCGAAGGCCAG
pJN105-lasR ΔV171	From pJN105L FWD	AGCAAACCGGTGGTTCTGAC
	From pJN105L REV	CGGATGTTCGAAGGCCAGTC
pJN105-lasR ΔS172	From pJN105L FWD	AAACCGGTGGTTCTGACCAG
	From pJN105L REV	GACCGGATGTTCGAAGGCCAG
pJN105-lasR ΔK173	From pJN105L FWD	CCGGTGGTTCTGACCAGCCG

	From pJN105L REV	GCTGACCGGATGTTCGAAGGC
pJN105-lasR Δ169, 171	From pJN105L FWD	CCGAGCAAACCGGTGGTTCTGACC
	From pJN105L REV	TTCGAAGGCCAGTCCGGCAC
pJN105-lasR Δ172-173	From pJN105L FWD	CCGGTGGTTCTGACCAGCCG
	From pJN105L REV	GACCGGATGTTCGAAGGCCAG
pJN105-lasR Δ169, 171-172	From pJN105L FWD	CCGAAACCGGTGGTTCTG
	From pJN105L REV	TTCGAAGGCCAGTCC
pJN105-lasR Δ170-172	From pJN105L FWD	AAACCGGTGGTTCTG
	From pJN105L REV	ATGTTCGAAGGCCAG
pJN105-lasR Scr 169-174	From pJN105L FWD	CCCGTCCACGTGGTTCTGACCAGC
	From pJN105L REV	TTTAGGCGATTCGAAGGCCAGTCC
pJN105-lasR Δ4-161	From pJN105L FWD	TCGAAGGCCAGTCCGGCACCCAAGGCCATAGCGC TACGTT
	From pJN105L REV	AACGTAGCGCTATGGCCTTGGGTGCCGGACTGGC CTTCGA
pJN105-lasR Δ4-168	From pJN105L FWD	ACCGGTTTGCTGACCGGATGCAAGGCCATAGCGC TACGTT
	From pJN105L REV	AACGTAGCGCTATGGCCTTGCATCCGGTCAGCAA ACCGGT
pJN105-FLAG-lasR Δ4-161	FLAG-LasR Δ4-161 FWD	GATGATGATAAAGGTGCCGGACTGGC
	FLAG-LasR Δ4-161 REV	ATCTTTATAATCCAAGGCCATAGCGCTAC
pJN105-FLAG-lasR Δ4-168	FLAG-LasR Δ4-168 FWD	GATGATGATAAACATCCGGTCAGCAAAC
	FLAG-LasR Δ4-168 REV	ATCTTTATAATCCAAGGCCATAGCGC
pJN105-Myc-LasR	From pJN105L FWD	AGCGAAGAAGATCTGGCCTTGGTTGACGGTTTTC
	From pJN105L REV	AATCAGTTTCTGTTCCATAGCGCTACGTTCTTC
pJN105-HA-lasR	From pJN105L FWD	TGCCGGATTATGCGGCCTTGGTTGACGGTTTTC
	From pJN105L REV	CATCATACGGATACATAGCGCTACGTTCTTC
pJN105-NLS-lasR	From pJN105L FWD	AACGCAAAGTGGCCTTGGTTGACGGTTTTC
	From pJN105L REV	TTTTTTTCGGCATAGCGCTACGTTCTTC
pJN105-His6-lasR	From pJN105L FWD	CACCACCACGCCTTGGTTGACGGTTTTC
	From pJN105L REV	ATGATGATGCATAGCGCTACGTTCTTC
pJN105-Gly6-lasR	From pJN105L FWD	GGTGGTGGTGGTGGCGCCTTGGTTGACGGTTTTC

	From pJN105L REV	ACCCATAGCGCTACGTTCTTC
pJN105-lasR Δ2-10	From pJN105L FWD	GAACGCTCAAGTGGAAAATTG
	From pJN105L REV	CATAGCGCTACGTTCTTC
pJN105-esaR +5: 165-169	From pJN105-esaR FWD	AACGAGCCGAAGGTGAACGAGCCCCGG
	From pJN105-esaR REV	CACCTTCGGTGCCTGCCAGGCGGTA
pJN105-esaR +5: 171-175	From pJN105-esaR FWD	ATCAGAGCGCGTTAAATCAGAGCGCG
	From pJN105-esaR REV	TTAACGCCGGGGCTCGTTCACCTTC
pJN105-esaR +5G at 171	From pJN105-esaR FWD	GTGGTGGCTTAAATCAGAGCGCGGACAAAAC
	From pJN105-esaR REV	CGCCACCCGCCGGGGCTCGTTC
pJN105-esaR +5G at 176	From pJN105-esaR FWD	GTGGTGGCGACAAAACGATATTTTCCTC
	From pJN105-esaR REV	CGCCACCCGCGCTCTGATTTAAC
pJN105-esaR +5A at 171	From pJN105-esaR FWD	CAGCCGCAGCGTTAAATCAGAGCGCG
	From pJN105-esaR REV	CCGCTGCCGGGGCTCGTTCACC
pJN105-esaR +5A at 176	From pJN105-esaR FWD	CAGCCGCAGCGGACAAAACGATATTTTC
	From pJN105-esaR REV	CCGCTGCGCTCTGATTTAACGCC
pJN105-esaR +10: 170-179	From pJN105-esaR FWD	CCGATAAAACGCCGGCGTTAAATCAG
	From pJN105-esaR REV	CTGATTGATTAAGGGCCGGGGCTCGTTCACCTTC
pJN105-esaR +20: (170-179)*2 Scr	From pJN105-esaR FWD	CAAGCCGCCACCAAGGACGCCCCGGCGTTAAATCAG
	From pJN105-esaR REV	ATTGGGAAGGTTGTCCTTAGATGGCTGCGTAGACAGCGCGGCTCGTTCACCTTC
pJN105-esaR +21: 159-179	Vector from pJN105-esaR FWD	CCACCGCGGTGGAGCTCCAATTCGCCCTATAGTGAGTCGT
	Vector REV	TCGGTGCCTGCCAGGCGGTACGTTTTGTCCGCGCTCTGAT
	Insert (from pJN105-esaR) FWD	ATCAGAGCGCGGACAAAACGTACCGCCTGGCAGGCACCGA

	Insert REV	ACGACTCACTATAGGGCGAATTGGAGCTCCACCG CGGTGG
pJN105-esaR Δ164-167	From pJN105-esaR FWD	CGAGCCCCGGCGTTAAATC
	From pJN105-esaR REV	GCCTGCCAGGCGGTAC
pJN105-esaR Δ168-171	From pJN105-esaR FWD	TTAAATCAGAGCGCGG
	From pJN105-esaR REV	TTCACCTTCGGTGCC
pJN105-esaR Δ172-175	From pJN105-esaR FWD	GCGGACAAAACGATATTTTCCTCG
	From pJN105-esaR REV	CGCCGGGGCTCGTTC
pJN105-esaR Δ176-179	From pJN105-esaR FWD	ATATTTTCCTCGCGTG
	From pJN105-esaR REV	GCTCTGATTTAACGCC
pJN105-esaR Δ170-173	From pJN105-esaR FWD	CAGAGCGCGGACAAAACG
	From pJN105-esaR REV	GGCTCGTTCACCTTCGGTG
pJN105-esaR Δ164-169	From pJN105-esaR FWD	CCGGCGTTAAATCAGAGCG
	From pJN105-esaR REV	GCCTGCCAGGCGGTAC
pJN105-esaR Δ171-176	From pJN105-esaR FWD	GACAAAACGATATTTTCCTCGC
	From pJN105-esaR REV	CGGGGCTCGTTCACCTT
pJN105-esaR Δ168-169, 171-176	From pJN105-esaR FWD	CCGGACAAAACGATATTTTCCTCGCG
	From pJN105-esaR REV	TTCACCTTCGGTGCCTGC
pJN105-esaR Δ164-171	From pJN105-esaR FWD	TTAAATCAGAGCGCGG
	From pJN105-esaR REV	GCCTGCCAGGCGGTAC
pJN105-esaR Δ164-175	From pJN105-esaR FWD	GCGGACAAAACGATATTTTCCTCG
	From pJN105-esaR REV	GCCTGCCAGGCGGTAC

pJN105-esaR scr 172-177	From pJN105-esaR FWD	GCGAGCAATAAAACGATATTTTCCTCGCGTGAAAATGAGG
	From pJN105-esaR REV	TAACTGGTCCGCCGGGGCTCGTTCAC
pJN105-esaR ΔY159	From pJN105-esaR FWD	CGCCTGGCAGGCACCGAA
	From pJN105-esaR REV	CATCTGCTCGTTAAAATCAATCAGCAGCATCTG
pJN105-esaR Δ159-162	From pJN105-esaR FWD	GGCACCGAAGGTGAACGAG
	From pJN105-esaR REV	CATCTGCTCGTTAAAATCAATCAGC
pJN105-esaR Y159A	From pJN105-esaR FWD	CAGGCACCGAAGGTGAACGAG
	From pJN105-esaR REV	CCAGGCGCGCCATCTGCTC
pJN105-esaR Y159F	From pJN105-esaR FWD	CGAGCAGATGTTTCGCCTGGCAG
	From pJN105-esaR REV	TTAAAATCAATCAGCAGCATCTG
pJN105-esaR Y159Q	From pJN105-esaR FWD	CAGGCACCGAAGGTGAACGAG
	From pJN105-esaR REV	CCAGGCGCTGCATCTGCTC
pJN105-esaR G163A	From pJN105-esaR FWD	CACCTTCGGTGGCTGCCAGGCGG
	From pJN105-esaR REV	CCGCCTGGCAGCCACCGAAGGTG
pJN105-esaR T164A	From pJN105-esaR FWD	GTTCACCTTCGGCGCCTGCCAGGCG
	From pJN105-esaR REV	CGCCTGGCAGGCGCCGAAGGTGAAC
pJN105-esaR E165A	From pJN105-esaR FWD	GGCTCGTTCACCTGCGGTGCCTGCCAG
	From pJN105-esaR REV	CTGGCAGGCACCGCAGGTGAACGAGCC
pJN105-esaR G166A	From pJN105-esaR FWD	GGGGCTCGTTCAGCTTCGGTGCCTG
	From pJN105-esaR REV	CAGGCACCGAAGCTGAACGAGCCCC
pJN105-esaR E167A	From pJN105-esaR FWD	GCCGGGGCTCGTGCACCTTCGGTGC

	From pJN105-esaR REV	GCACCGAAGGTGCACGAGCCCCGGC
pJN105-esaR R168A	From pJN105-esaR FWD	GCCGGGGCTGCTTCACCTTCGGTGCCTG
	From pJN105-esaR REV	CAGGCACCGAAGGTGAAGCAGCCCCGGC
pJN105-esaR P170A	From pJN105-esaR FWD	TCTGATTTAACGCCGCGGCTCGTTCACCTTC
	From pJN105-esaR REV	GAAGGTGAACGAGCCGCGGCGTTAAATCAGA
pJN105-esaR L172A	From pJN105-esaR FWD	CCGCGCTCTGATTTGCCGCCGGGGCTCGTT
	From pJN105-esaR REV	AACGAGCCCCGGCGGCAAATCAGAGCGCGG
pJN105-esaR N173A	From pJN105-esaR FWD	CCGCGCTCTGAGCTAACGCCGGGGCTCG
	From pJN105-esaR REV	CGAGCCCCGGCGTTAGCTCAGAGCGCGG
pJN105-esaR Q174A	From pJN105-esaR FWD	GTCCGCGCTCGCATTTAACGCCGGGGCTCG
	From pJN105-esaR REV	CGAGCCCCGGCGTTAAATGCGAGCGCGGAC
pJN105-esaR S175A	From pJN105-esaR FWD	TATCGTTTTGTCCGCGGCCTGATTTAACGCCGGG G
	From pJN105-esaR REV	CCCCGGCGTTAAATCAGGCCGCGGACAAAACGAT A
pJN105-esaR D177A	From pJN105-esaR FWD	GAGGAAAATATCGTTTTGGCCGCGCTCTGATTTAA CG
	From pJN105-esaR REV	CGTTAAATCAGAGCGCGGCCAAAACGATATTTTCC TC
pJN105-esaR K178A	From pJN105-esaR FWD	ACGCGAGGAAAATATCGTTGCGTCCGCGCTCTGA TTTAAC
	From pJN105-esaR REV	GTTAAATCAGAGCGCGGACGCAACGATATTTTCCT CGCGT
pJN105-esaR T179A	From pJN105-esaR FWD	CGAGGAAAATATCGCTTTGTCCGCGCTCTGATTTA AC
	From pJN105-esaR REV	GTTAAATCAGAGCGCGGACAAAGCGATATTTTCCT CG
pJN105-esaR I180A	From pJN105-esaR FWD	ATTTTCACGCGAGGAAAATGCCGTTTTGTCCGCGC TCTGA
	From pJN105-esaR REV	TCAGAGCGCGGACAAAACGGCATTTTCCTCGCGT GAAAAT

pJN105-esaR F4A	From pJN105-esaR FWD	AACCAAACAATAACGGATACG
	From pJN105-esaR REV	TTCAAGGAACGCAGAGAACATG
pJN105-esaR F5A	From pJN105-esaR FWD	AACCAAACAATAACGGATACG
	From pJN105-esaR REV	TTCAAGCGCGAAAGAGAACATG
pJN105-mrtR +4G at 171	From pJN105-mrtR FWD	GGTGGTGAGAACGATCCGGTCC
	From pJN105-mrtR REV	GCCACCCCCGTGCAACTCGTAAAC
pJN105-mrtR +4G at 175	From pJN105-mrtR FWD	GGTGGTGTCCCCGCCCTTTCG
	From pJN105-mrtR REV	GCCACCCGGATCGTTCTCCCC
pJN105-mrtR +4A at 171	From pJN105-mrtR FWD	Resulted from cloning of pJN105-MrtR +5A at 171
	From pJN105-mrtR REV	
pJN105-mrtR +4A at 175	From pJN105-mrtR FWD	GCGGCAGTCCCCGCCCTTTCG
	From pJN105-mrtR REV	CGCTGCCGGATCGTTCTCCCC
pJN105-mrtR +5A at 171	From pJN105-mrtR FWD	CAGCGGCAGAGAACGATCCGGTCC
	From pJN105-mrtR REV	CCGCTGCCCCGTGCAACTCGTAAAC
pJN105-mrtR +5A at 175	From pJN105-mrtR FWD	CAGCGGCAGTCCCCGCCCTTTCG
	From pJN105-mrtR REV	CCGCTGCCGGATCGTTCTCCCC
pJN105-mrtR ∆171-173	From pJN105-mrtR FWD	GATCCGGTCCCCGCCCTT
	From pJN105-mrtR REV	GTGCAACTCGTAAACCGCTTTGC
pJN105-expR2 +5A at 171	From pJN105-expR2 FWD	CAGCGGCAAGAAAACAAGGTGACAAAGAG
	From pJN105-expR2 REV	CCGCTGCGCTCTGCGGGCTTTG
pJN105-expR2 +5A at 176	From pJN105-expR2 FWD	CAGCGGCAAAAGAGATTTTCTCCCAG

	From pJN105-expR2 REV	CCGCTGCGTCACCTTGTTTTCTGC
pJN105-expR2 +5G at 171	From pJN105-expR2 FWD	GTGGTGGCAGAAAACAAGGTGACAAAGAG
	From pJN105-expR2 REV	CGCCACCGCTCTGCGGGCTTTG
pJN105-expR2 +5G at 176	From pJN105-expR2 FWD	GTGGTGGCAAAGAGATTTTCTCCCAG
	From pJN105-expR2 REV	CGCCACCGTCACCTTGTTTTCTGC
pJN105-expR2 +5: 172-176	From pJN105-expR2 FWD	GGTGACAAAGAGATTTTCTCCCAGCG
	From pJN105-expR2 REV	TTGTTTTCTGTCACCTTGTTTTCTGCTCTG
pJN105-expR2 +10: 168-177	From pJN105-expR2 FWD	AAGGAGACAAAAGCCCGCAGAGCAGAAAAC
	From pJN105-expR2 REV	GTTTACGACTTTGAGGTGATTGCGTCATCTCTTTG TAAAGCG
pJN105-expR2 +21:158-178	From pJN105-expR2 FWD	ACGTAAGCAAGGAGACAAAGAGATCACCACGCTT TACAAAG
	From pJN105-expR2 REV	GACTGCGGTGATTGTGTCATTTCCTTATACAAAGT AGTAATTTTCTCATGAACGGCAATG
pJN105-expR2 Δ169-172	From pJN105-expR2 FWD	AAACAAGGTGACAAAGAG
	From pJN105-expR2 REV	GCTTTGCGTCATCTCT
pJN105-expR2 Δ173-176	From pJN105-expR2 FWD	AAAGAGATTTTCTCCCAGCGC
	From pJN105-expR2 REV	TCTGCTCTGCGGGCTTTG
pJN105-expR2 Δ170-175	From pJN105-expR2 FWD	GACAAAGAGATTTTCTCCCAGCG
	From pJN105-expR2 REV	CGGGCTTTGCGTCATCTCTTTG
pJN105-expR2 Δ169-176	From pJN105-expR2 FWD	AAAGAGATTTTCTCCCAGCG
	From pJN105-expR2 REV	GCTTTGCGTCATCTCTTTGTAAAG
pJN105-expR2 K177A	From pJN105-expR2 FWD	ACAAGGTGACGCGGAGATTTTCTCCCAG
	From pJN105-expR2 REV	TTTCTGCTCTGCGGG

pJN105-expR2 E178A	From pJN105-expR2 FWD	AGGTGACAAAGCGATTTTCTCCC
	From pJN105-expR2 REV	TGTTTTCTGCTCTGCG
pJN105-expR2 F4A	From pJN105-expR2 FWD	CAATGAAATTATCAATAACACGAT
	From pJN105-expR2 REV	TCAGAGCACGCTACAGACAT
pJN105-expR2 Δ2-175	From pJN105-expR2 FWD	GACAAAGAGATTTTCTCCC
	From pJN105-expR2 REV	CATGAATTCGCTAGCC
pJN105-FLAG-expR2 Δ2-177	From pJN105-expR2 FWD	GATGATGATAAAGAGATTTTCTCCCAGC
	From pJN105-expR2 REV	ATCTTTATAATCCATGAATTCGCTAGCC

<u>Raw western blot images.</u>

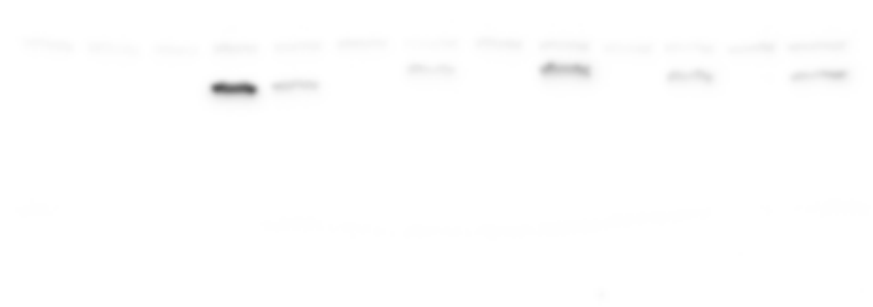

Lanes 3-13 correspond to FLAG blot shown on left in **Figure S5E**.

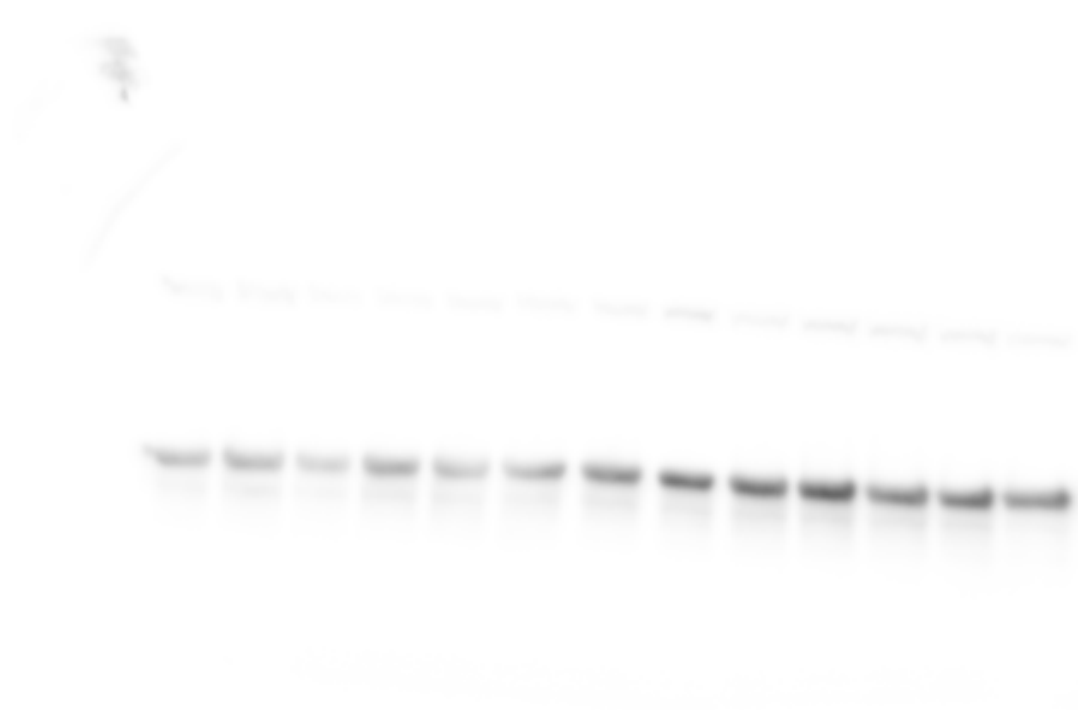

Lanes 3-13 correspond to GAPDH blot shown on left in **Figure S5E**.

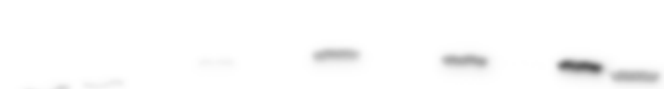

Lanes 1-11 correspond to FLAG blot shown on left of **Figure S12A**.

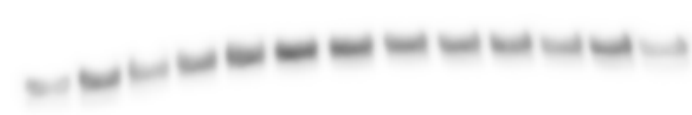

Lanes 1-11 correspond to GAPDH blot shown on left of **Figure S12A**.

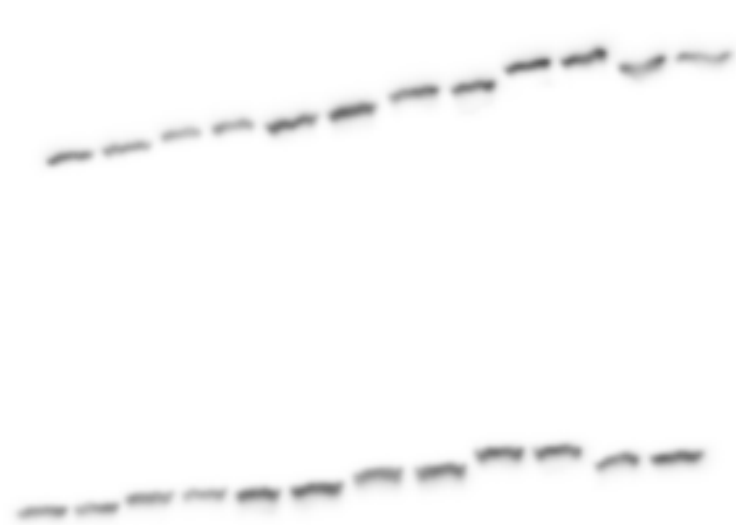

Two gels with separate biological replicates were transferred to same membrane and imaged together. Top corresponds to FLAG for **Figure S12B**. Bottom corresponds to FLAG for **Figure S8B**.

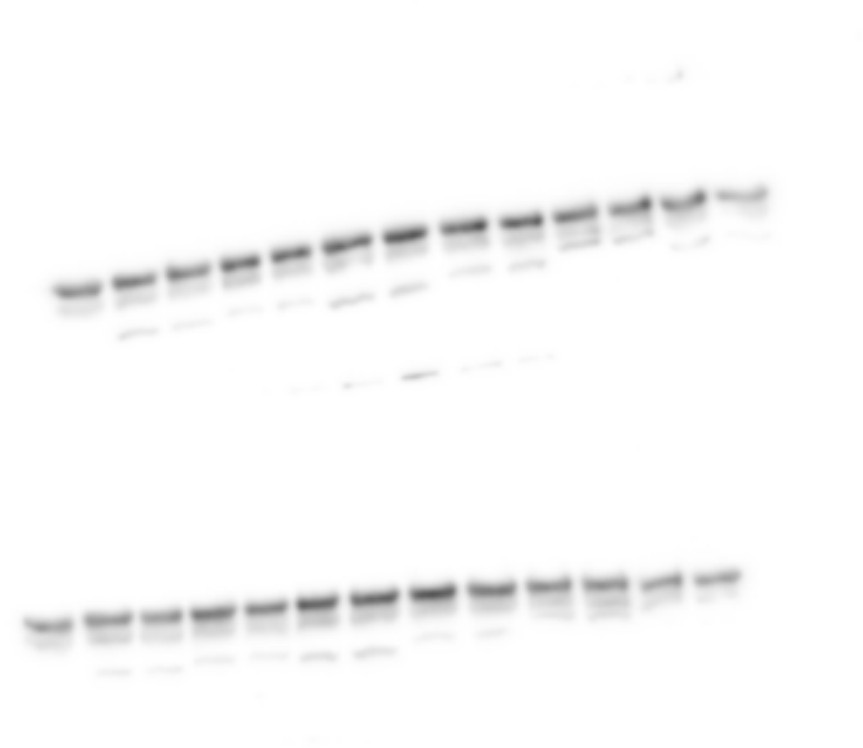

Two gels with separate biological replicates transferred to same membrane and imaged together. Top corresponds to GAPDH for **Figure S12B**. Bottom corresponds to GAPDH for **Figure S8B**. The corresponding FLAG blot shown above was treated with 10% acetic acid for 30 min at 37 °C to inactivate HRP, then washed in water and TBST and re-stained for GAPDH.

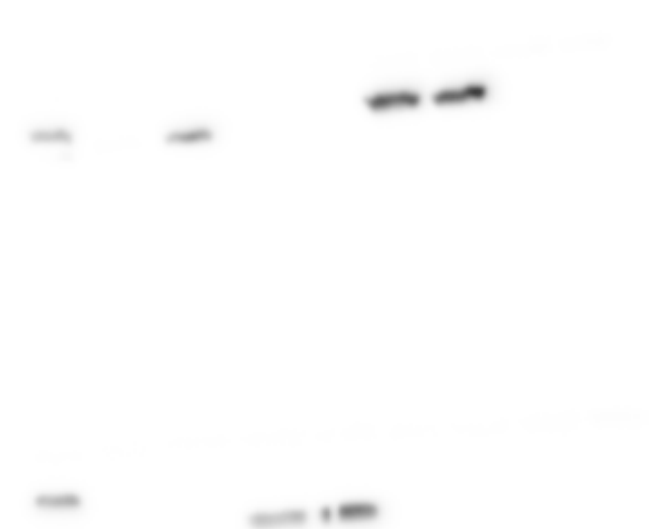

Top row, lanes 4-11 correspond to FLAG blot shown in middle of **Figure S5E**. Bottom row, lanes 4-13 correspond to FLAG blot shown on right in **Figure S5E**.

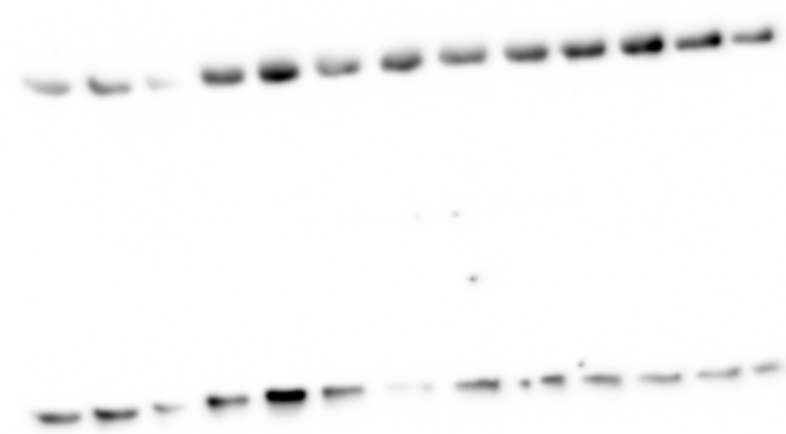

Top row, lanes 4-11 correspond to GAPDH blot shown in middle of **Figure S5E**. Bottom row, lanes 4-13 correspond to GAPDH blot shown on right in **Figure S5E**. The corresponding FLAG blot shown above was treated with 10% acetic acid for 30 min at 37 °C to inactivate HRP, then washed in water and TBST and stained for GAPDH.

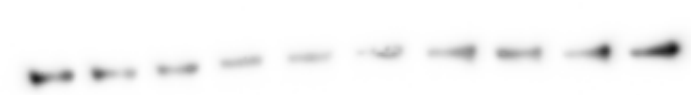

GAPDH blot using the same samples as the GAPDH blot shown on right of **Figure S5E** and directly above, which showed uneven staining.

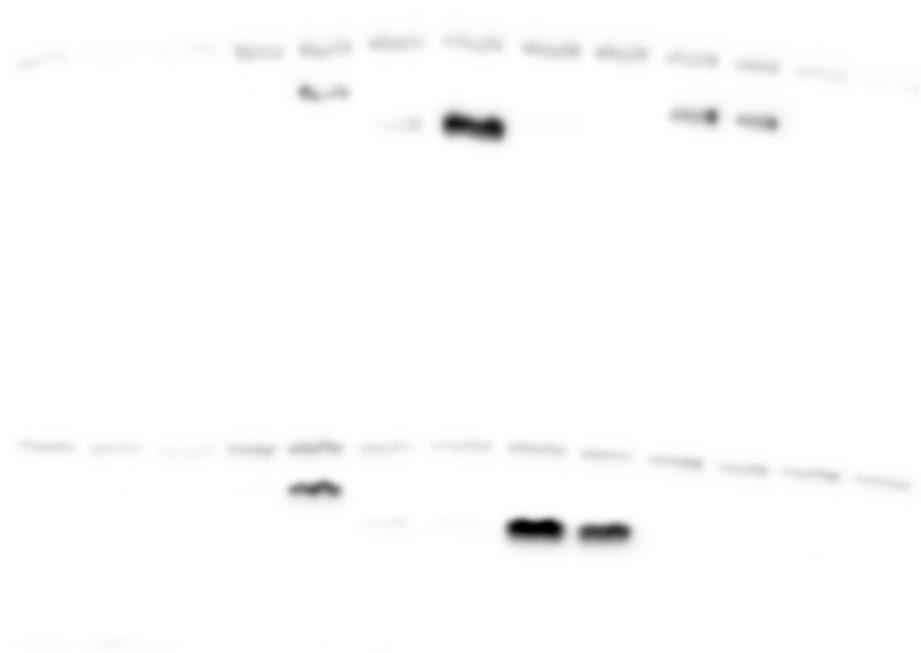

Top row, lanes 4-11 correspond to FLAG blot shown in middle of Figure **S12A**. Bottom row, lanes 4-13 correspond to FLAG blot shown on right of Figure **S12A**.

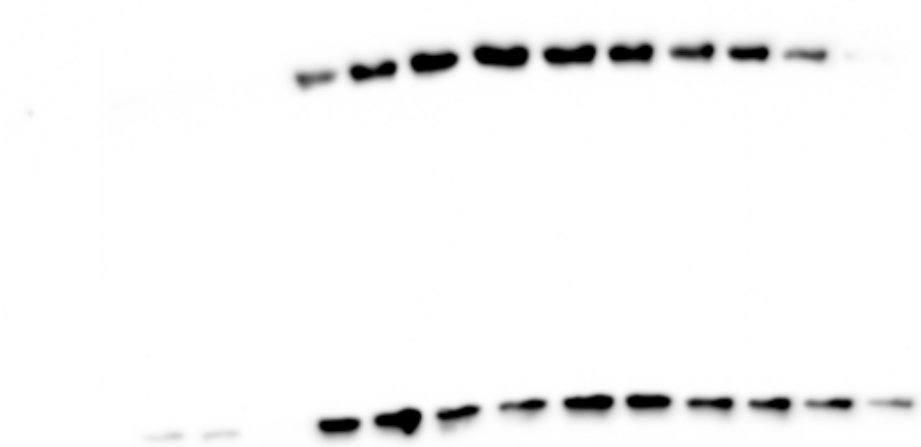

Top row corresponds to FLAG blot shown in middle of **Figure S12A**. Bottom row, lanes 4-13 correspond to FLAG blot shown on right of **Figure S12A**. Lanes 1-3 are very faint in both blots. The corresponding FLAG blot shown directly above was treated with 10% acetic acid for 30 min at 37 °C to inactivate HRP, then washed in water and TBST and stained for GAPDH.

References.

(1) Sievers, F.; Wilm, A.; Dineen, D.; Gibson, T. J.; Karplus, K.; Li, W.; Lopez, R.; McWilliam, H.; Remmert, M.; Söding, J.; et al. Fast, scalable generation of high-quality protein multiple sequence alignments using Clustal Omega. *Mol. Syst. Biol.* **2011**, *7*, 539. DOI: https://doi.org/10.1038/msb.2011.75.

(2) Madeira, F.; Madhusoodanan, N.; Lee, J.; Eusebi, A.; Niewielska, A.; Tivey, A. R. N.; Lopez, R.; Butcher, S. The EMBL-EBI Job Dispatcher sequence analysis tools framework in 2024. *Nucleic Acids Res.* **2024**, *52* (W1), W521–W525. DOI: https://doi.org/10.1093/nar/gkae241.

(3) Le, N. T. P.; Phan, T. T. P.; Phan, H. T. T.; Truong, T. T. T.; Schumann, W.; Nguyen, H. D. Influence of N-terminal His-tags on the production of recombinant proteins in the cytoplasm of *Bacillus subtilis*. *Biotechnol. Rep. (Amst)* **2022**, *35*, e00754. DOI: https://doi.org/10.1016/j.btre.2022.e00754.

(4) Köppl, C.; Buchinger, W.; Striedner, G.; Cserjan-Puschmann, M. Modifications of the 5' region of the CASPONTM tag's mRNA further enhance soluble recombinant protein production in *Escherichia coli*. *Microb. Cell Fact.* **2024**, *23* (1), 86. DOI: https://doi.org/10.1186/s12934-024-02350-z.

(5) Slater, H.; Crow, M.; Everson, L.; Salmond, G. P. Phosphate availability regulates biosynthesis of two antibiotics, prodigiosin and carbapenem, in *Serratia* via both quorum-sensing-dependent and -independent pathways. *Mol. Microbiol.* **2003**, *47* (2), 303–320. DOI: https://doi.org/10.1046/j.1365-2958.2003.03295.x.

(6) Cui, Y.; Chatterjee, A.; Hasegawa, H.; Chatterjee, A. K. *Erwinia carotovora* subspecies produce duplicate variants of ExpR, LuxR homologs that activate *rsmA* transcription but differ in their interactions with *N*-acylhomoserine lactone signals. *J. Bacteriol.* **2006**, *188* (13), 4715–4726. DOI: https://doi.org/10.1128/jb.00351-06.

(7) Schu, D. J.; Carlier, A. L.; Jamison, K. P.; von Bodman, S.; Stevens, A. M. Structure/function analysis of the *Pantoea stewartii* quorum-sensing regulator EsaR as an activator of transcription. *J. Bacteriol.* **2009**, *191* (24), 7402–7409. DOI: https://doi.org/10.1128/jb.00994-09.

(8) Kim, Y.; Chhor, G.; Tsai, C. S.; Fox, G.; Chen, C. S.; Winans, N. J.; Jedrzejczak, R.; Joachimiak, A.; Winans, S. C. X-ray crystal structures of the pheromone-binding domains of two quorum-hindered transcription factors, YenR of *Yersinia enterocolitica* and CepR2 of *Burkholderia cenocepacia*. *Proteins* **2017**, *85* (10), 1831–1844. DOI: https://doi.org/10.1002/prot.25336.

(9) Lamb, J. R.; Patel, H.; Montminy, T.; Wagner Victoria, E.; Iglewski Barbara, H. Functional domains of the RhlR transcriptional regulator of *Pseudomonas aeruginosa*. *J. Bacteriol.* **2003**, *185* (24), 7129–7139. DOI: https://doi.org/10.1128/JB.185.24.7129-7139.2003.

(10) Lee, J.-H.; Lequette, Y.; Greenberg, E. P. Activity of purified QscR, a *Pseudomonas aeruginosa* orphan quorum-sensing transcription factor. *Mol. Microbiol.* **2006**, *59* (2), 602–609. DOI: https://doi.org/10.1111/j.1365-2958.2005.04960.x.

(11) Poulter, S.; Carlton, T. M.; Spring, D. R.; Salmond, G. P. The *Serratia* LuxR family regulator CarR 39006 activates transcription independently of cognate quorum sensing signals. *Mol. Microbiol.* **2011**, *80* (4), 1120–1131. DOI: https://doi.org/10.1111/j.1365-2958.2011.07634.x.

(12) Stevens, A. M.; Queneau, Y.; Soulère, L.; von Bodman, S.; Doutheau, A. Mechanisms and synthetic modulators of AHL-dependent gene regulation. *Chem. Rev.* **2011**, *111* (1), 4–27. DOI: https://doi.org/10.1021/cr100064s.

(13) Welsh, M. A.; Blackwell, H. E. Chemical probes of quorum sensing: from compound development to biological discovery. *FEMS Microbiol. Rev.* **2016**, *40* (5), 774–794. DOI: https://doi.org/10.1093/femsre/fuw009.

(14) Palmer, A. G.; Mukherjee, A.; Stacy, D. M.; Lazar, S.; Ané, J.-M.; Blackwell, H. E. Interkingdom responses to bacterial quorum sensing signals regulate frequency and rate of nodulation in legume–rhizobia symbiosis. *ChemBioChem* **2016**, *17* (22), 2199–2205. DOI: https://doi.org/10.1002/cbic.201600373.

(15) Lindsay, A.; Ahmer, B. M. Effect of *sdiA* on biosensors of *N*-acylhomoserine lactones. *J. Bacteriol.* **2005**, *187* (14), 5054–5058. DOI: https://doi.org/10.1128/jb.187.14.5054-5058.2005.

(16) Khlebnikov, A.; Datsenko, K. A.; Skaug, T.; Wanner, B. L.; Keasling, J. D. Homogeneous expression of the P_{BAD} promoter in *Escherichia coli* by constitutive expression of the low-affinity high-capacity AraE transporter. *Microbiology* **2001**, *147* (12), 3241–3247. DOI: https://doi.org/10.1099/00221287-147-12-3241.

(17) Chugani, S. A.; Whiteley, M.; Lee, K. M.; D'Argenio, D.; Manoil, C.; Greenberg, E. P. QscR, a modulator of quorum-sensing signal synthesis and virulence in *Pseudomonas aeruginosa*. *Proc. Natl. Acad. Sci. U. S. A.* **2001**, *98* (5), 2752–2757. DOI: https://doi.org/10.1073/pnas.051624298.

(18) Stoutland, I. M.; Aguirre-Figueroa, G.; Blackwell, H. E. Chemical probes that target a dissociative LuxR-type quorum sensing receptor in gram-negative bacteria. *ACS Chem. Biol.* **2025**, *20* (10), 2451–2461. DOI: https://doi.org/10.1021/acschembio.5c00490.

(19) Schuster, M.; Urbanowski, M. L.; Greenberg, E. P. Promoter specificity in *Pseudomonas aeruginosa* quorum sensing revealed by DNA binding of purified LasR. *Proc. Natl. Acad. Sci. U. S. A.* **2004**, *101* (45), 15833–15839. DOI: https://doi.org/10.1073/pnas.0407229101.
